# Genetic deletion of zinc transporter ZnT_3_ induces progressive cognitive deficits in mice by impairing dendritic spine plasticity and glucose metabolism

**DOI:** 10.3389/fnmol.2024.1375925

**Published:** 2024-05-14

**Authors:** Rui Zong, Xiaoding Zhang, Xiaohui Dong, Guan Liu, Jieyao Zhang, Yiting Gao, Zhongyang Zhang, Yiming Ma, Haixia Gao, Nikita Gamper

**Affiliations:** ^1^Department of Pharmacology, Center for Innovative Drug Research and Evaluation, Institute of Medical Science and Health, The Hebei Collaboration Innovation Center for Mechanism, Diagnosis and Treatment of Neurological and Psychiatric Disease, The Key Laboratory of Neural and Vascular Biology, Ministry of Education, Hebei Medical University, Shijiazhuang, Hebei, China; ^2^Faculty of Biological Sciences, School of Biomedical Sciences, University of Leeds, Leeds, United Kingdom

**Keywords:** ZnT_3_, zinc, dendritic plasticity, spatial memory, glycometabolism

## Abstract

Zinc transporter 3 (ZnT_3_) is abundantly expressed in the brain, residing in synaptic vesicles, where it plays important roles in controlling the luminal zinc levels. In this study, we found that ZnT_3_ knockout in mice decreased zinc levels in the hippocampus and cortex, and was associated with progressive cognitive impairments, assessed at 2, 6, and 9-month of age. The results of Golgi-Cox staining demonstrated that ZnT_3_ deficiency was associated with an increase in dendritic complexity and a decrease in the density of mature dendritic spines, indicating potential synaptic plasticity deficit. Since ZnT_3_ deficiency was previously linked to glucose metabolism abnormalities, we tested the expression levels of genes related to insulin signaling pathway in the hippocampus and cortex. We found that the Expression of glucose transporters, GLUT3, GLUT4, and the insulin receptor in the whole tissue and synaptosome fraction of the hippocampus of the ZnT_3_ knockout mice were significantly reduced, as compared to wild-type controls. Expression of AKT (A serine/threonine protein kinase) and insulin-induced AKT phosphorylation was also reduced in the hippocampus of ZnT_3_ knockout mice. We hypothesize that the ZnT_3_ deficiency and reduced brain zinc levels may cause cognitive impairment by negatively affecting glycose metabolism via decreased expression of key components of insulin signaling, as well as via changes in synaptic plasticity. These finding may provide new therapeutic target for treatments of neurodegenerative disorders.

## Introduction

There is increasing evidence that zinc transport proteins are crucial for numerous biological processes, particularly those depending on or regulated by zinc, including zinc-dependent enzymatic reactions, ion channel regulation, cell division and differentiation, synaptic transmission, and many others (Takeda, 2014). Two major families of zinc transporters were identified in mammals, ZIPs (ZRT, IRT-like Proteins) and ZnTs (Zinc Transporters), both of which encompass solute carrier proteins (SLCs) (Kambe et al., 2014). Generally, ZIPs transport zinc into the cytosol and ZnTs work in the opposite direction (Kambe et al., 2014). There are currently 10 members of ZnT family, ZnT1-10, with ZnT_3_ being identified as a neuronal member, localizing to synaptic membranes (Kimura and Kambe, 2016). ZnT_3_ (SLC30A3) was cloned and characterized in 1996 (Palmiter et al., 1996); the gene is predominantly found in the cerebral cortex and hippocampus, where it influences neurogenesis in the adult hippocampus and contributes to spatial learning and memory behaviors (Choi et al., 2020).

ZnT_3_ facilitates the entry of cytoplasmic zinc into vesicles containing glutamate, accordingly, zinc is co-released into the synaptic cleft during neuronal excitation (Martel et al., 2011; Choi et al., 2020). ZnT_3_ is actually one of the most abundant membrane proteins found in synaptic vesicles in the brain (Upmanyu et al., 2022). While exact role of ZnT_3_ in the nervous system is only beginning to emerge, it was linked to memory and memory deficits. Thus, it was observed that late-stage dementia patients exhibited a notable decrease in the ZnT_3_ expression in the cortical regions, as compared to the control group (Beyer et al., 2009). This finding may suggest that the impairment of neuronal ability to process Zn^2+^ could potentially be a casual factor in the development of dementia. This conclusion aligns with the discovery indicating that the sequestration of zinc within oligomeric Aβ-Zn complexes could potentially diminish zinc accessibility at the synapse, consequently, this reduction in zinc may contribute to cognitive impairments in individuals with Alzheimer's disease (AD) (Deshpande et al., 2009).

Multiple studies have demonstrated an association between Type 2 Diabetes mellitus (T2DM) and cognitive decline (Pal et al., 2018; Xue et al., 2019; Alsharif et al., 2020). Such decline in cognitive function can be attributed to the impaired absorption of glucose in neurons, which hampers energy production. Notably, glucose transporters, such as GLUT3 and GLUT4 are present in the blood-brain barrier and are highly expressed in hippocampus. Downregulation of GLUT3, GLUT4, and insulin receptors, leading to disturbances in glucose transport and utilization has been reported in AD patients (Kandimalla et al., 2017). AKT (A serine/threonine protein kinase) is a key mediator of insulin signaling and loss of AKT expression and/or activity may lead to insulin resistance and glucose tolerance (Cho et al., 2001; Garofalo et al., 2003; Manning and Cantley, 2007). Loss-of-function Akt mutations have been found in patients with severe insulin resistance, while gain-of-function mutations may cause hypoglycemia (George et al., 2004; Hussain et al., 2011). Hence, there are emerging similarities in some key cellular and molecular mechanisms between diabetes and insulin resistance associated with cognitive decline (including the AD) in elderly (Kandimalla et al., 2017).

Dendritic spines serve as the principal loci for excitatory neurotransmission in the mature brain. The malfunction and degeneration of synapses significantly contribute to the pathogenesis and advancement of AD. Interestingly, led-induced reduction of GLUT4 plasma membrane abundance in hippocampal neurons was shown to impair hippocampal glucose metabolism and reduce dendritic spine formation, resulting in learning and memory deficits (Zhao et al., 2021).

Global ZnT_3_ knockout mouse line (ZnT${\;}_{3}^{-/-}$) was generated in the Palmiter's laboratory (Cole et al., 1999) and these were shown to demonstrate some cognitive deficits and decreased synaptic spine density in the hippocampus (Vogler et al., 2022). Here, we used ZnT${\;}_{3}^{-/-}$ mice to investigate importance of ZnT_3_ for cognitive functions, synaptic plasticity and expression of key glucose transporters during mouse development. Our findings reveal that ZnT_3_ deficiency is associated with numerous age- and sex-related spatial memory deficits, synaptic plasticity abnormalities, reduced expression of GLUT3, GLUT4, and insulin receptors in the hippocampus and a disrupted insulin signaling pathway. These findings reveal strong association between synaptic zinc transport, glucose metabolism and cognitive function.

## Materials and methods

### Animals

All animal experiments were performed in accordance with the Animal Care and Ethical Committee of Hebei Medical University (approval number: IACUC-Hebmu-2020007). Male and female *Slc30a3* knock-out (ZnT${\;}_{3}^{-/-}$) mice (Jackson Laboratories, JAX stock #005064) were utilized in this study. These mice express a cassette containing nuclear lacZ and neo, which replaces exon 1 through 4 (Cole et al., 1999). At the age of 3 weeks, genotyping of was performed using polymerase chain reaction (PCR) of tail DNA, following the protocols provided by Jackson Laboratories. C57/BL6 mice were used as WT controls.

### Insulin treatment

Mice were split into four cohorts: (i) Control WT mice (WT control); (ii) WT mice receiving insulin glargine (a prolonged action insulin formulation; 160337-95-1, MedChemExpress, USA) (WT insulin); (iii) Control ZnT${\;}_{3}^{-/-}$ mice (ZnT${\;}_{3}^{-/-}$ control); (iv) ZnT${\;}_{3}^{-/-}$ mice receiving insulin glargine (ZnT${\;}_{3}^{-/-}$ insulin). Mice were fasted for 5 h, insulin glargine was administered to insulin-recieving groups intraperitoneally (i.p) at 2 IU/kg; saline (0.9%, w/v) was used as a vehicle in the control groups (Fang et al., 2017). After 24 h all animals were sacrificed, brain tissue samples were then promptly collected for analysis.

### Timm-Danscher Zn^2+^ labeling

Mice were deeply anesthetized with isoflurane and perfused with a 0.3% sodium sulfide solution, followed by saline and 4% paraformaldehyde (PFA). The brains were isolated and fixed with 4% PFA, and then dehydrated with 30% sucrose. The fresh-frozen tissue was sectioned using cryostat (Leica CM1950, Germany) at 25 μm thickness. These slices were placed in a developing solution at 26°C, and then immersed in a 5% sodium metabisulfite solution for 10 min to halt the metal self-developing reaction. Following dehydration and permeabilization, the slices were placed on glass microscope slides, sealed with neutral tree gum and observed using an optical microscope (Olympus BX63, Japan). The developer solution was prepared according to sodium sulfide Timm's method (Danscher, 1996).

### TSQ [N- (6-methoxy-8-quinolyl)-4-toluenesulfonamide] staining

The staining was performed as described previously (Kiyohara et al., 2021). Briefly, mice were deeply anesthetized and sacrificed; brains were promptly excised and flash-frozen in liquid nitrogen. The brain sections were prepared in the same way as for Timm-Danscher staining. These sections were subjected to a 2-min wash with a PBS solution. TSQ (ATT Bioquest, USA; 100 μM in ddH2O) was then applied onto the sections for 30 min. The sections were then washed with PBS, placed on glass microscope slides and sealed with cover slips. Immunofluorescence was visualized using a confocal laser microscopy system (Olympus FV1200MPE, Japan), with 360 nm excitation. The 5 mM TSQ stock solution was prepared in DMSO and diluted to the working concentration with ddH_2_O, the working solution also contained of sodium acetate and barbital, both at a concentration of 140 mM.

### Golgi-Cox staining and analysis

For the Golgi-Cox staining we utilized the commercially available kit (FD Rapid GolgiStainTM Kit #PK401), following the protocol provided by the manufacturer. The mouse brains were immersed in the impregnation solution (solution A/B of the kit) for 3 weeks, the tissues were then sectioned at 150 μm using a vibratome (Leica VT1200S, Germany). These sections were then placed into the staining solution (solution D/E of the kit) and subjected to conventional dehydration and penetration sequence, according to the kit's protocol. The sections were then placed on the glass slides and sealed with neutral gum. Imaging was performed using on the Olympus BX63 microscope with 100x oil-immersion objective. Z-stacks were collected to reconstruct the neurons. The images were subsequently analyzed using ImageJ plugin (Sholl); the analysis was performed manually. Only the cells exhibiting complete cell bodies with dendrites that appeared dark brown or black, without any observable signs of dendrite severance were selected for analysis. Dendritic segments that were located on second-or third-order dendrites were observed (Stranahan et al., 2009).

### Immunofluorescence

The brain sections were prepared in the same way as for Timm-Danscher staining. Following incubation with 0.3% Triton X-100 at 37°C for 1 h, sections were blocked with 10% donkey serum (SL050, Solaobio, China) or goat serum (BMS0050, Abbkine, USA) at either 37°C or room temperature for 1 h. Subsequently, sections were incubated with the primary antibodies overnight at 4°C, followed by three washes with PBS the following day. The secondary antibodies were then added and incubated at room temperature for 90 min. After three additional washes with PBS, the sections were incubated with DAPI (D9542, Sigma, USA; 1:200) for 10 min. Finally, the sections were mounted on the glass slides and sealed using a cover slip. The primary antibodies used, along with their respective dilution standards, were as follows: rabbit anti-ZnT_3_ polyAb antibody (17363-1-AP, Proteintech, USA; 1:200); rabbit anti-GLUT3 ployclonal antibody (20403-1-AP, Proteintech, USA; 1:200); mouse anti-GLUT4 monoclonal antibody (66846-1-lg, Proteintech, USA; 1:200); rabbit anti-NeuN monoclonal antibody (ET1602-12, HUABIO, China; 1:200); mouse anti-GFAP antibody (610565, Pharmingen, USA; 1:200); rabbit anti-Akt (pan) (C67E7, Cell Signaling, USA; 1:200); and rabbit anti-phospho-Akt (Ser 473) (D9E, Cell Signaling, USA; 1:200). The secondary antibodies were donkey anti-mouse Alexa Fluor 488 (A21202, Invitrogen, USA; 1:500); donkey anti-rabbit Alexa Fluor 568 (A10042, Invitrogen, USA; 1:500); and goat anti-rabbit Alexa Fluor 594 (A11012, Invitrogen, USA; 1:500). Immunofluorescence was visualized and analyzed using a confocal laser microscopy system (ZEISS LSM900 Airyscan2, Germany; 3D HISTECH Pannoramic SCAN, Hungary).

## Behavioral tests

### Novel object recognition and novel location recognition tests

These experiments consisted of three distinct phases undertaken with 24 h intervals (Chao et al., 2020). Novel object recognition (NOR) tests were performed as follows: during the initial phase (habituation), the mice were given the opportunity to freely explore an opaque chamber containing two spatial clue objects (plastic shapes), referred to as O1 and O2, for 10 min. Next day, the second phase (training) involved placing the same objects in the box and allowing the mice to freely explore the entire environment for another 10 min. On the third day (the novel object recognition, NOR phase), the object O2 was replaced with one of different color and shape (referred to as “O3”), and the mice were once again given 10 min to freely explore. The movement of the animals was recorded using the Smart 3.0 System (Panlab, Spain), and the results were analyzed by the operator unaware of the genotype of mice tested. The interaction time (T) was measured with Smart System, and the preference index was defined as T_O3_/(T_O3_+ T_O2_).

Novel location recognition (NLR) tests were performed in the same or similar chambers as follows: during the initial habituation, the mice were exploring an opaque box containing four spatial clue objects: O1–O4 for 10 min. Next day, the second phase (training) involved placing the same objects in the box in the same positions and allowing the mice to freely explore the entire environment for another 10 min. On the next day (novel location recognition, NLR phase), the positions of O2 and O3 in the box were swapped, and the mice were allowed to explore the entire environment and object for 10 min. The experiments were recorded and analyzed the same way as in the NOR test. The interaction time (T) was defined as (T_O3_+T_O2_)/(T_O1_+T_O2_+T_O3_+T_O4_).

### Spontaneous alternation test (Y-maze)

The experimental protocol entailed a singular 5-min trial wherein the mice were allowed unrestricted navigation and exploration of all three arms of the Y-maze (5 × 35 × 40 cm). To ensure the trial's integrity, mice attempting to scale the maze walls were promptly and gently returned to the arm it had exited. Random assignment of start arms was implemented to mitigate potential bias in start arm placement. The operator was unaware of test mice genotype. The memory index was determined by tallying the sequential entries into the three distinct arms (designated as A, B, and C), and subsequently dividing this value by the overall potential alternations (the total number of arm entries −2) (Hughes, 2004).

### Morris water maze test

The Morris Water Maze Test was conducted in accordance with the experimental protocol by Oz et al. (2017), the test assesses the mice's capacity for memory retention. The Morris Water Maze system comprised of a water tank with dimensions of 90 cm in diameter and 50 cm in height, filled with water (20–24°C) (Tian et al., 2019). A platform measuring 8 × 8 cm was concealed 2 cm beneath the water surface. To enhance visibility, the water was tinted white using titanium dioxide. The water tank was partitioned into four quadrants. Spatial navigation experiments were conducted on mice over the course of the initial 5 days, an animal was granted a 60s period to navigate the maze each time. The duration spent in the platform, the number of entries into the platform, and the swimming velocity of the mice were documented. On the 6^th^ day, a spatial exploration experiment was performed, druing which the platform was removed, and the duration spent at the place previousely occupied by the platform, as well as the number of entries into that space were analised. The operator was unaware of the test animal genotype.

### Open field test

The open field test was executed in accordance with the protocol by Lin and Hsueh (2014). The apparatus employed was identical to the NOR/NLR tests. The central square area was established to be equivalent in size to the combined area of the four corners of the open field. At the onset of the experiment, the mice were individually positioned at the center of the enclosure, and their locomotor activities were captured via top-view video recordings for a duration of 5 min. The Smart System was utilized to measure both the overall distance covered by the mice and the distance confined within the central region. The operator was unaware of the test animal genotype.

### T-maze reversal learning test

The test is based on the methodology outlined in Shih et al. (2020). The animals were individually housed and subjected to dietary restrictions for 1 week, body weight of the mice was reduced to 80%−85% of original body weight. A food reward was positioned at the end of one of the goal arms of the T-maze (approach alley: 71 × 10 × 10 cm; goal arms: 46 × 10 × 10 cm). The mice were trained as follows: during each training days 10 trials were performed. Within each trial, a mouse was placed in a designated area at the conclusion of the approach alley, enabling unrestricted exploration of the maze. One of the two arms contained a reward. When the mice selected the reward-containing arm, they were permitted to consume food for a duration of 40 seconds. Conversely, if the mice opted for the empty arm, no form of reward or punishment was administered. Once the mice's accuracy in choosing the reward-containing arm surpassed or equaled 80% for a continuous period of 3 days, they were subjected to reverse learning on the subsequent day. In the course of reverse learning, the food pellet was relocated to the opposite direction. The duration of time required for the mice to attain 80% accuracy in choosing the new reward-containing arm was documented. The operator was unaware of the test animal genotype.

### Measurement of plasma glucose

Blood glucose concentrations were measured using a glucometer (Yuwell, China). Fasting was implemented for each group of mice starting at 8:00 in the morning to detect blood glucose levels during fasting. To investigate the effects of fasting on mouse blood glucose, mice were briefly anesthetized with isoflurane and tail vein blood collected at the time intervals of 0 h, 2 h, 4 h, and 6h after the onset of fasting. Prior to fasting, all mice received *ad libitum* food access (Andrikopoulos et al., 2008).

### Real-time qPCR

The total RNA extracted from the cortex and hippocampus was evaluated for concentration and purity using spectrophotometry. Total RNA was converted into cDNA using the HiScript III RT SuperMix kit (Vazyme, China). The PCR amplification was performed using specific primer sets (Supplementary Table 1) and the qPCR was performed using ChamQ Universal SYBR qPCR Master Mix kit (Vazyme, China), following the manufacturer's instructions. The qPCR analysis involved an initial denaturation step at 95°C for 30 s, followed by 40 cycles of denaturation at 95°C for 5 s and annealing at 60°C for 30 s, and final dissociation step at 72°C for 30 s. qPCR data analysis was performed using the 2^−Δ*ΔCT*^ method.

### Membrane (synaptosome) protein isolation

The synaptosome proteins in the whole brain of mice were obtained using MinuteTM Synaptosome Isolation Kit (SY-052, Invent Biotechnologies, Minnesota, USA), according to the manufacturer's instructions (Ke et al., 2023). Briefly, filter cartridges were pre-chilled on ice; brain tissue samples (20–30 mg) were transferred into the cartridges and 200 μl of ice-cold buffer A added. Tissue was homogenized with a plastic rod and further 300 μl buffer A added. High-speed centrifugation (16,000 g, 15–20s) was performed, the precipitate suspension was vortexed for 10 seconds, followed by medium-speed (1,500 g, 5 min) centrifugation, and the supernatant was transferred to a new tube. After adding 50 μl of ice-cold buffer B and mixing, high-speed centrifugation (13,000 g, 15 min) was carried out to thoroughly remove the supernatant (cytoplasmic components). Pellets were resuspended in ice-cold buffer C (500 μl), incubated on ice for 5 min, followed by medium-speed (2,000 g, 5 min) centrifugation; supernatant was collected and centrifuged at 13,000 g (20 min), to obtain “synaptosome” precipitate.

### Western blotting

Tissue samples were lysed with RIPA buffer (32010A, BestBio, China) supplemented with protease (HY-K0010, Sigma-Aldrich, USA) and phosphatase inhibitors (G2007, Servicebio, China) for 30 min on ice. The samples were centrifuged at 13,400 g for 20 min at 4°C and the supernatant containing proteins was removed. The proteins in samples underwent size-fractionation on a 10% SDS-PAGE gel and were subsequently transferred onto a polyvinylidene fluoride (PVDF) (0.45 μm) membrane. The membranes were then blocked in Tris buffered saline with Tween (TBST) solution containing 5% milk powder for 2 h. The membranes were incubated overnight with primary antibodies diluted in TBST. The following primary antibodies were used: rabbit anti-GLUT3 ployclonal antibody (20403-1-AP, Proteintech, USA; 1:2,000); mouse anti-GLUT4 monoclonal antibody (66846-1-lg, Proteintech, USA; 1:2,000); rabbit anti-GSK-3β monoclonal antibody (D5C5Z, Cell Signaling, USA; 1:1,000); and anti-GAPDH recombinant rabbit monoclonal antibody (SA30-01, HUABIO, China; 1:5,000). Next day the membranes were washed and incubated with anti-rabbit IgG (H+L) (DyLight 800 Conjugate) (515S, Cell Signaling, USA; 1:5,000) or anti-mouse IgG (H+L) (DyLight 800 Conjugate) (5257S, Cell Signaling, USA; 1:5,000). After washing with TBST the PVDF membranes were incubated with Western LightningTM Chemiluminescence Reagent (NEL10300EA, PerkinElmer, USA) for 30 seconds, and covered with photosensitive film for 1 min. The PVDF membranes were then incubated in Stripping buffer (SW3020, Solarbio, China) for 30 min, and steps were repeated to obtain the GAPDH staining.

### Statistical analysis

For comparison of two groups of data, an independent-samples *t-*test was used; if the data did not meet the assumptions of the parametric test, such as normality or equal variance, the Mann-Whitney test was used. One or two-way ANOVA with LSD *post hoc* test was used to compare the means of multiple groups. After *post-hoc* tests, significance level of *p* < 0.05 adopted as signifying statistically significant difference. The statistical analysis was performed using GraphPad Prism 9. The data are reported as mean ± standard error (S.E).

## Results

### Deletion of ZnT_3_ reduces brain zinc level

We used ZnT${\;}_{3}^{-/-}$ mice in which the initial four exons of ZnT_3_ are substituted with a construct comprising a nuclear *lacZ (nlacZ)* cassette and a neomycin-resistant gene (*neo*^*r*^) driven by a *polII* promoter (Cole et al., 1999) (Supplementary Figure 1A). Successful deletion of *Slc30a3* (ZnT_3_) was confirmed by qPCR and immunofluorescence (Supplementary Figures 1B–E). In accord with previous findings, labeling synaptic zinc using TSQ and Timm's method (Danscher, 1996; Cole et al., 1999; Meeusen et al., 2011; Choi et al., 2017) revealed drastically reduced abundance of zinc in the hippocampus (especially in the CA3 region) of ZnT${\;}_{3}^{-/-}$ mice, as compared to WT controls (Supplementary Figures 1F, G). There was also marked decrease of synaptic zinc in the cortex (Supplementary Figure 1E).

### Deletion of ZnT_3_ in mice results in spatial memory deficits and cognitive inflexibility

**Figure 1:**
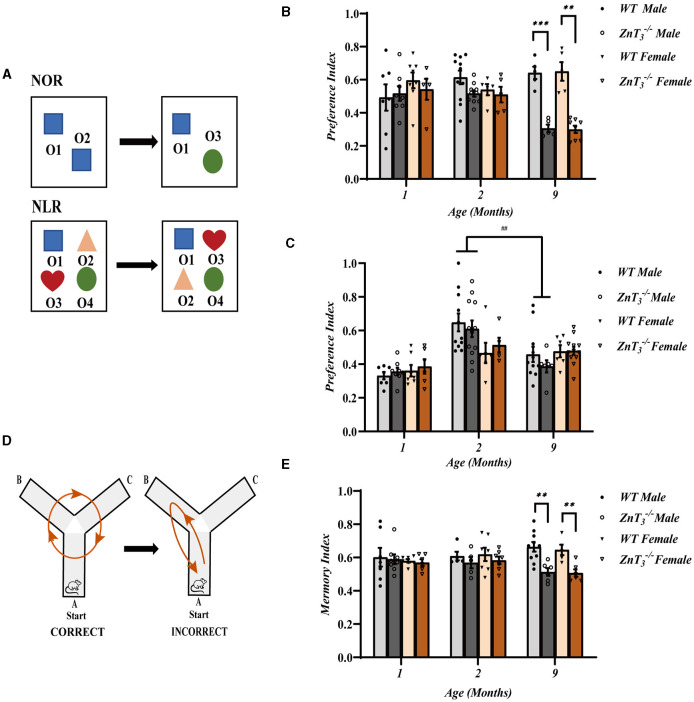
Knockout of ZnT_3_ in mice is associated with spatial memory deficit and cognitive inflexibility. **(A)** Schematic diagram of novel object recognition (NOR) and novel location recognition (NLR) tests; O1, O2, O3, and O4 designate plastic blocks of different shape used as objects. **(B, C)** The results of NOR **(C)** and NLR **(D)** tests performed on mice of 1-, 2-, and 9-months of age. Data are expressed as the preference index; NOR preference index: [T_O3_/(T_O3_+ T_O2_)] (1 Month WT males *n* = 7; 1 Month ZnT${\;}_{3}^{-/-}$ males, *n* = 8; 1 Month WT females, *n* = 8; 1 Month ZnT${\;}_{3}^{-/-}$ females, *n* = 5; 2 Month WT males, *n* = 11; 2 Month ZnT${\;}_{3}^{-/-}$ males, *n* = 10; 2 Month WT females, *n* = 5; 2 Month ZnT${\;}_{3}^{-/-}$ females, *n* = 5; 9 Month WT males, *n* = 5; 9 Month ZnT${\;}_{3}^{-/-}$ males, *n* = 5; 9 Month WT females, *n* = 5; 9 Month ZnT${\;}_{3}^{-/-}$ females, *n* = 8); NLR preference index: [(T_O3_+T_O2_)/(T_O1_+T_O2_+T_O3_+T_O4_)] (1 Month WT males, *n* = 7; 1 Month ZnT${\;}_{3}^{-/-}$ males, *n* = 8; 1 Month WT females, *n* = 7; 1 Month ZnT${\;}_{3}^{-/-}$ females, *n* = 6; 2 Month WT males, *n* = 11; 2 Month ZnT${\;}_{3}^{-/-}$ males, *n* = 12; 2 Month WT females, *n* = 6; 2 Month ZnT${\;}_{3}^{-/-}$ females, *n* = 5; 9 Month WT males, *n* = 11; 9 Month ZnT${\;}_{3}^{-/-}$ males, *n* = 6; 9 Month WT females, *n* = 6; 9 Month ZnT${\;}_{3}^{-/-}$ females, *n* = 12). Here and everywhere mean data are presented as mean ± S.E.M. **, *** and ^##^ Denote significant difference between groups indicated by the connector lines, *p* < 0.01 or *p* < 0.001, two-way ANOVA with LSD *post-hoc* test. **(D)** Schematic diagram of the Y-maze test (spontaneous alteration test). **(E)** The result of Y-maze test. Data are expressed as the memory index [the number of alternations/(the total number of arm entries −2)] (1 Month WT males, *n* = 7; 1 Month ZnT${\;}_{3}^{-/-}$ males, *n* = 8; 1 Month WT females, *n* = 7; 1 Month ZnT${\;}_{3}^{-/-}$ females, *n* = 6; 2 Month WT males, *n* = 5; 2 Month ZnT${\;}_{3}^{-/-}$ males, *n* = 5; 2 Month WT females, *n* = 6; 2 Month ZnT${\;}_{3}^{-/-}$ females, *n* = 7; 9 Month WT males, *n* = 10; 9 Month ZnT${\;}_{3}^{-/-}$ males, *n* = 6; 9 Month WT females, *n* = 5; 9 Month ZnT${\;}_{3}^{-/-}$ females, *n* = 6). ** Denote significant difference between groups indicated by the connector lines, *p* < 0.01, two-way ANOVA with LSD *post-hoc* test.

We employed multiple behavioral paradigms to assess cognitive function of ZnT_3_ knockout mice. To evaluate recognition of novelty, we performed novel object recognition (NOR) and novel location recognition (NLR) tests on 1, 2, and 9-month-old animals (Figure 1A). No significant difference between the genotypes was observed in younger animals, at 2-month, male ZnT${\;}_{3}^{-/-}$ mice started to display a tendency toward reduced exploration of the novel object and at 9-month-old ZnT${\;}_{3}^{-/-}$ mice of either gender displayed dramatic reduction of the novel object exploration (Figure 1B). No significant difference between genotypes was observed in the NLR test, although male mice of both genotypes demonstrated an age-related decline in exploration of novelty in this test, which may indicate general spatial memory decline (Figure 1C). To further explore potential effects of ZnT_3_ deletion on spatial memory, we conducted a spontaneous alternation experiment using the Y-maze (Figure 1D). Consistent with the NOR test results, the preference index (time) was significantly reduced in 9-month-old ZnT_3_ knockout mice, regardless of gender (Figure 1E). Additionally, the results of the appetite-driven T maze experiment indicated that ZnT_3_ had no obvious impact on the mice's ability to identify and learn alternative locations of food (Supplementary Figure 2).

**Figure 2:**
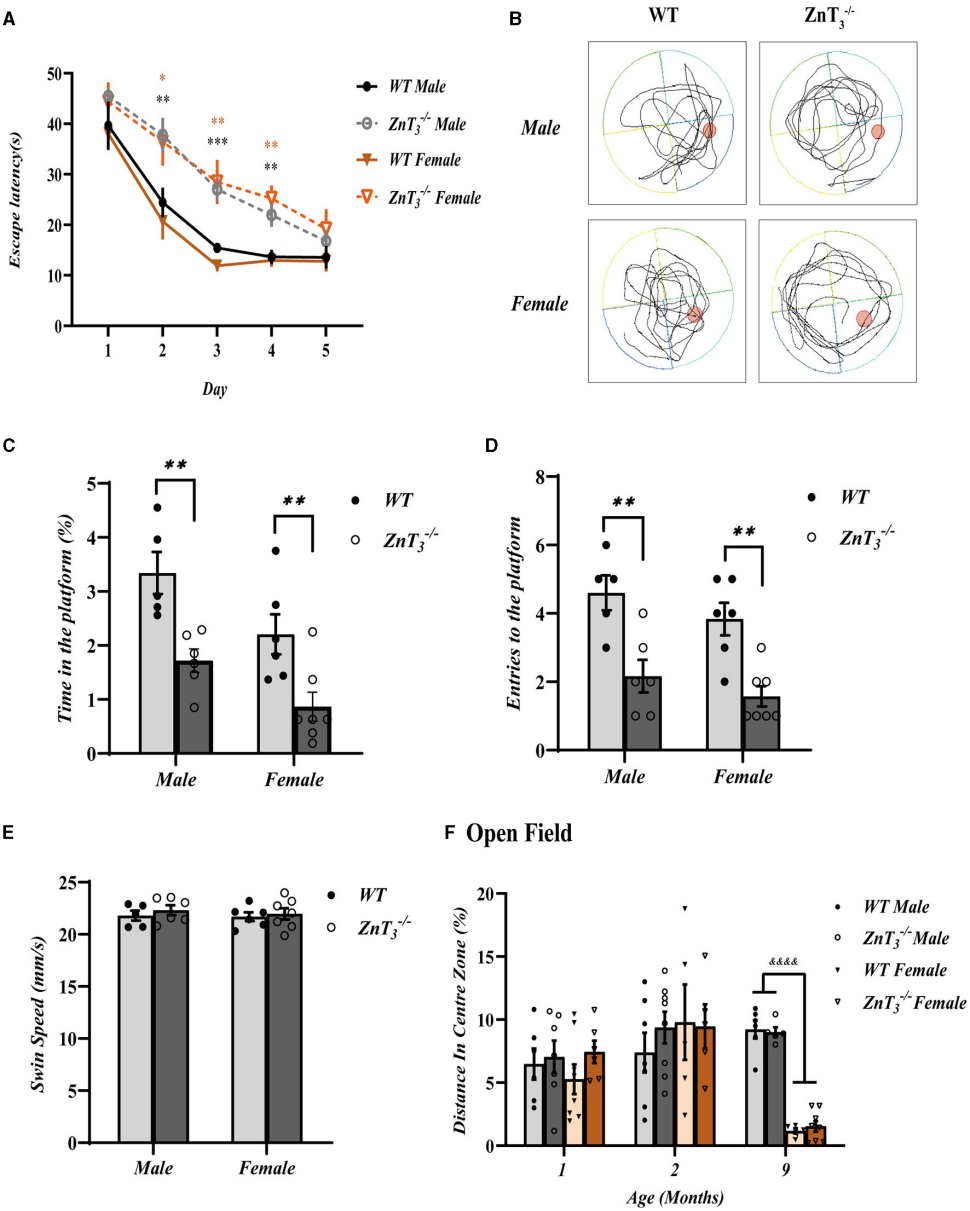
Knockout of ZnT_3_ in mice is associated with spatial reference memory deficit. **(A)** Morris water maze (MWM) performance. Escape latency during the acquisition trials for five consecutive days (WT males, *n* = 8; ZnT${\;}_{3}^{-/-}$ males, *n* = 9; WT females, *n* = 7; ZnT${\;}_{3}^{-/-}$ females, *n* = 9). *, **, *** Denote significant difference between time-matched WT and ZnT${\;}_{3}^{-/-}$ groups, *p* < 0.05, *p* < 0.01, or *p* < 0.001, two-way ANOVA with LSD *post-hoc* test. **(B)** Representative tracing images of swimming trajectories the MWM probe trial for male of female ZnT${\;}_{3}^{-/-}$ and WT mice. The position of the target platform is indicated with a red circle. **(C, D)** Analysis of the MWM tests (WT males, *n* = 5; ZnT${\;}_{3}^{-/-}$ males, *n* = 6; WT females, *n* = 6; ZnT${\;}_{3}^{-/-}$ females, *n* = 7). **(C)** Time spent within the area formerly occupied by the platform during the probe trial (as a percentage of the total trial time). **(D)** Counts of entries into the platform area during the probe trial. **(E)** Swim speed during the probe trial. ** Denotes significant difference between groups indicated by the connector lines, *p* < 0.01, two-way ANOVA with LSD *post-hoc* test. **(F)** The results of open field test, analyzed as distance traveled in the central zone (as percentage of total distance traveled). ^&&&&^ Denotes significant difference between groups indicated by the connector lines, *p* < 0.0001, two-way ANOVA with LSD *post-hoc* test.

The Morris Water Maze (MWM) experiments were then performed to assess the impact of ZnT_3_ deletion on the spatial learning abilities of mice. Swimming ability deteriorated with age (Yanai and Endo, 2021) and, hence, these experiments were only performed at 2-months-old mice. ZnT${\;}_{3}^{-/-}$ mice of both genders exhibited significantly prolonged escape latencies over the duration of training (5 days; Figure 2A). WT mice generally reached their minimum escape latencies by the 3^rd^ training day, while ZnT${\;}_{3}^{-/-}$ controls only approached this level of performance by 5^th^ day. Furthermore, when the platform was removed during the trial (day 6; Figure 2B), ZnT${\;}_{3}^{-/-}$ mice displayed significantly reduced time spent in the target quadrant and a decreased number of platform crossings (Figures 2C, D), indicating impaired spatial learning. The swimming speed was not affected by the ZnT_3_ knockout, indicating that ZnT${\;}_{3}^{-/-}$ mice did not exhibit any deficiencies in neuromuscular strength or exploratory locomotion (Figure 2E). The MWM experiment did not reveal any disparities between genders. Overall, the MWM experiments revealed significantly impaired spatial learning ability of ZnT${\;}_{3}^{-/-}$ mice of either gender.

The anxiety levels of mice in a novel environment were assessed using the open field test. The experiment revealed no difference between genotypes, however at 9 months of age female mice of either genotype exhibited shorter presence in the central zone, compared to males (Figure 2F). This observation may be attributed to the decrease in estrogen levels in elderly female mice, as suggested earlier (Xu et al., 2016).

The above findings indicate that ZnT_3_ deletion had a detrimental effect on spatial memory and learning and on cognitive flexibility, but did not lead to anxiety.

Analysis of behavioral outcomes revealed that there was no significant difference in spatial memory between female and male ZnT${\;}_{3}^{-/-}$ mice. Therefore, in the following histological experiments, a mixed group of male and female mice was used.

### ZnT_3_ deletion results in impaired dendritic plasticity in the hippocampus

**Figure 3:**
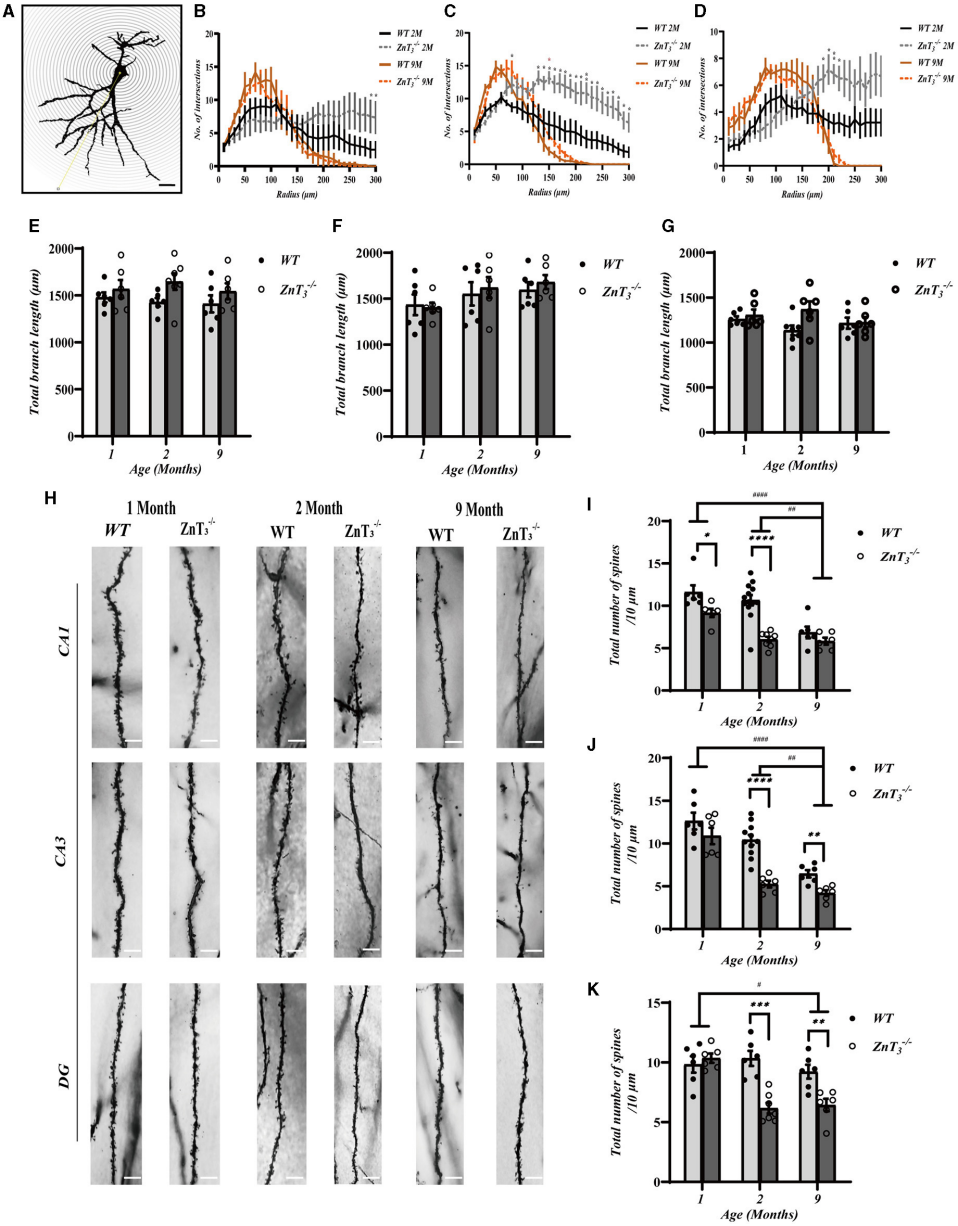
ZnT_3_ deletion is associated with increased dendrite complexity and reduced spine density in the hippocampus. **(A)** Diagram showing Sholl analyses to quantify dendrite length and complexity. Scale bar, 50 μm. **(B–D)** Statistical analysis for the number of intersections in CA1 **(B)**, CA3 **(C)** and DG **(D)** of hippocampus. Two- and 9-month-old mice of both genders were analyzed (WT 2 M group, *n* = 7; ZnT${\;}_{3}^{-/-}$ 2 M group, *n* = 6; WT 9 M group, *n* = 6; ZnT${\;}_{3}^{-/-}$ 9 M group, *n* = 6). *, ** Denote significant difference of number of intersections between radius (μm)-matched WT and ZnT${\;}_{3}^{-/-}$ groups, *p* < 0.05 or *p* < 0.01, two-way ANOVA with LSD *post-hoc* test. **(E–G)** Statistical analysis of the total dendritic length in CA1 **(E)**, CA3 **(F)**, and DG **(G)** of hippocampus. No significant difference was found (Two-way ANOVA with LSD *post-hoc* test). **(H)** Example Golgi staining images of dendritic spines in CA1, CA3 and DG of hippocampus of 1-month-old, 2-months-old, and 9-months-old mice. Scale bar, 5 μm. **(I–K)** Statistical analysis of dendritic spine density from images as these shown in H in CA1 **(I)**, CA3 **(J)**, and DG **(K)** regions of hippocampus. *, **, ***, **** and ^#^, ^##^, ^####^ Denote significant difference between groups indicated by the connector lines, *p* < 0.05, *p* < 0.01, *p* < 0.001, or *p* < 0.0001, two-way ANOVA with LSD *post-hoc* test.

To gather data on the potential alterations in the synaptic plasticity in the hippocampus, a Golgi-Cox staining of sections of hippocampus were subjected to Sholl analysis of dendritic complexity (Ferreira T. A. et al., 2014). This approach quantifies number of crossings processes make at different concentric distances form the cell body (Figure 3A). Irrespective of genotype, the number of intersections with the concentric circles was significantly lower in aged mice (9-month-old), as compared to young (2-month-old) (Figures 3B–D), which was consistent with previous findings (Duan et al., 2003). Interestingly, in comparison to WT controls, the 2-month-old ZnT${\;}_{3}^{-/-}$ mice exhibited an increase in the number of intersections in CA3 and DG of the hippocampus. These findings suggest that the dendritic arborization in these regions of hippocampus is more complex in the ZnT${\;}_{3}^{-/-}$ mice of the younger age (as the difference was no longer observed at the 9 months of age (Figures 3B–D). Total dendritic length generally did not change between genotypes (Figures 3E–G).

**Figure 4:**
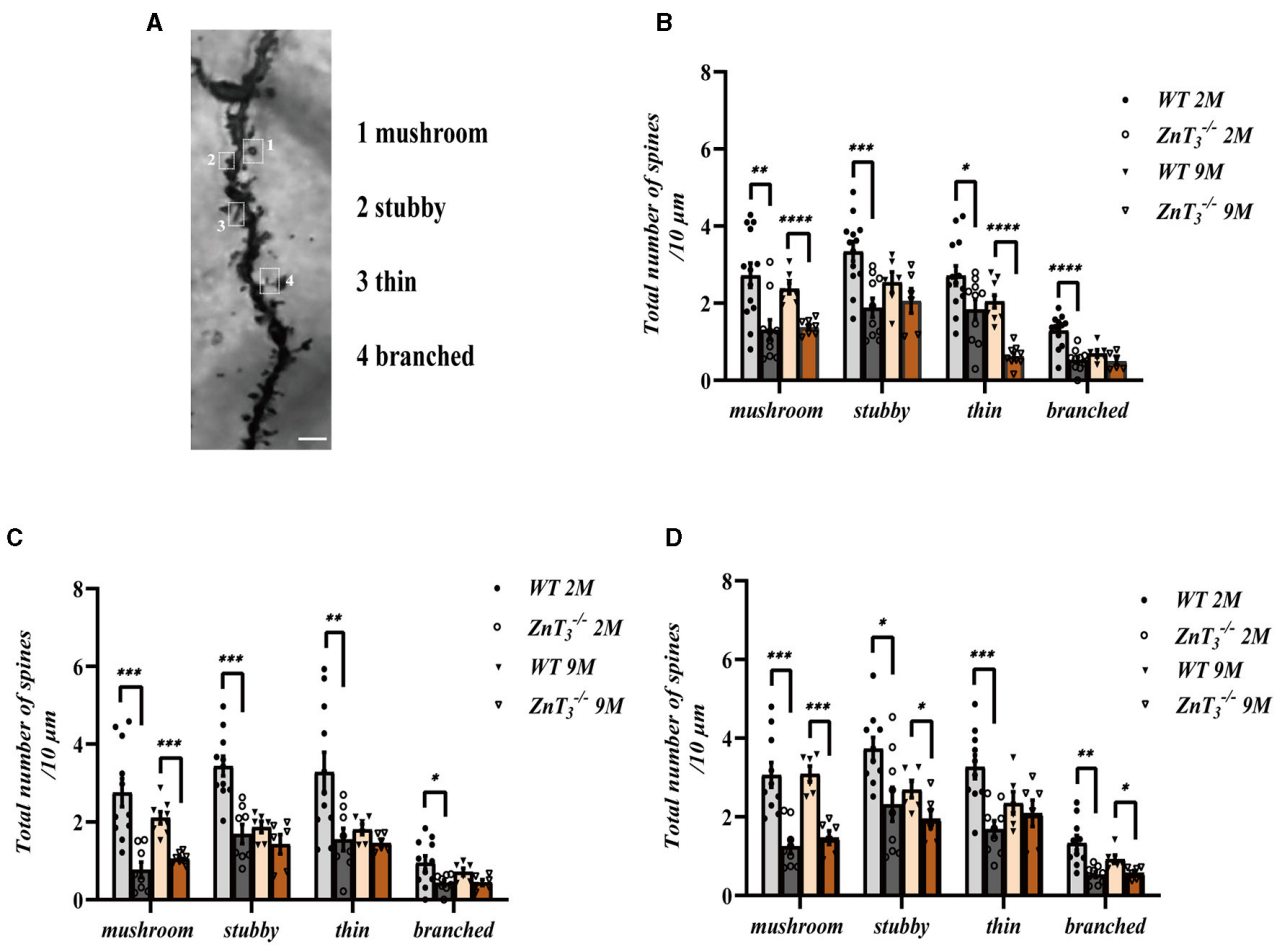
Analysis of the effect of ZnT_3_ knockout on the densities of different types of dendritic spines in the hippocampus. **(A)** Example image showing classification of spines. Scale bar, 2.5 μm. **(B–D)** Statistical analysis for Golgi staining images of different types of spines in CA1 **(B)**, CA3 **(C)**, and DG **(D)** regions of hippocampus. Two- and 9-month-old mice, male and female mice were analyzed. *, **, ***, **** Denote significant difference between groups indicated by the connector lines, *p* < 0.05, *p* < 0.01, *p* < 0.001, or *p* < 0.0001, two-way ANOVA with LSD *post-hoc* test.

We also examined the morphological alterations in dendritic spines of hippocampal neurons in ZnT${\;}_{3}^{-/-}$ mice at various developmental stages (Figure 3H). The results showed that there was a general decline in dendritic spine density with age in both, the WT and ZnT${\;}_{3}^{-/-}$ mice but the knockout animals displayed a more prominent decline (Figures 3I–K). Specifically, in the CA1 and DG regions of WT mice hippocampi, the total spine density was similar in 1- and 2-months-old animals and decreased to approximately half of that level by 9 months of age. In ZnT${\;}_{3}^{-/-}$ mice the maximal decline was already apparent at 2-months of age (significantly different from WT). In the CA3 region total synaptic spine density did not significantly change in the WT animals but did decline in ZnT${\;}_{3}^{-/-}$. Further analysis of the specific spine types (“mushroom,” “stubby,” “thin” and “branched”; Figure 4A) revealed that all four types of spines were decreased in density in 2-months-old ZnT${\;}_{3}^{-/-}$ mice (Figures 4B–D). We divided hippocampal CA1 neuronarborization into basal and apical dendrites and compared WT and ZnT${\;}_{3}^{-/-}$ mice (Supplementary Figure 3A). Both the basal and apical dendritic spine number in the CA1 region decreased with age in both, WT and ZnT${\;}_{3}^{-/-}$ mice. Generally, ZnT${\;}_{3}^{-/-}$ mice had lower spine densities at both, basal and apical dendrites (as compared to WT mice) with the most dramatic difference observed at 2-months of age (Supplementary Figures 3B, C). The densities of all four types of spines in basal and apical dendrites were significantly reduced in 2-months-old ZnT${\;}_{3}^{-/-}$ mice (Supplementary Figures 3D–G). These findings indicate that the elimination of ZnT_3_ had a detrimental effect on the hippocampal dendrite plasticity in mice.

### ZnT_3_ deletion is associated with reduced expression of key determinants of glycometabolism

**Figure 5:**
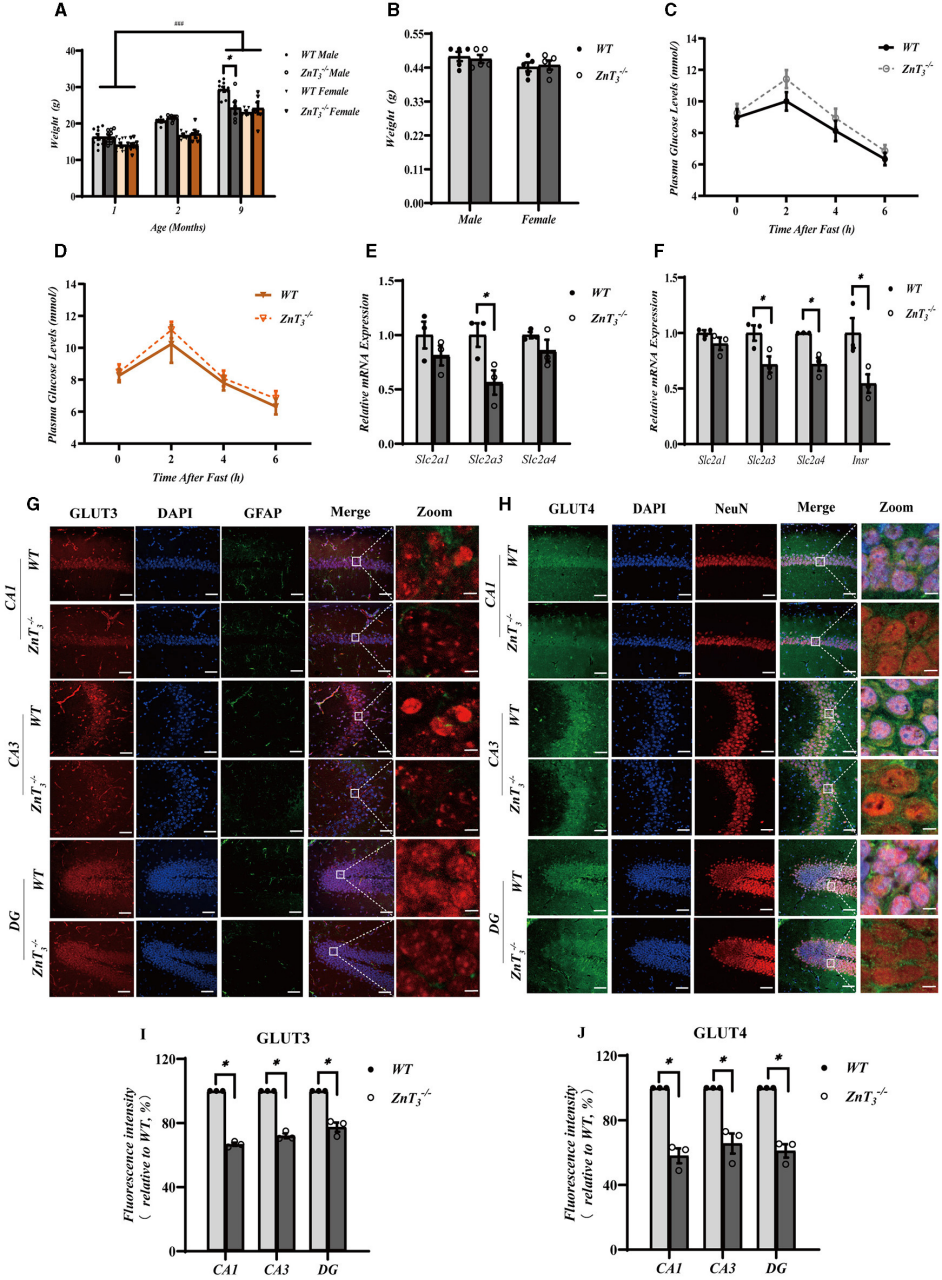
ZnT_3_ deletion is associated with a significant impairment of insulin signaling in the hippocampus. **(A)** Body mass analysis of ZnT${\;}_{3}^{-/-}$ and WT mice. *, ^###^ Denote significant difference between groups indicated by the connector lines, *p* < 0.05, *p* < 0.001, two-way ANOVA with LSD *post-hoc* test. **(B)** Brain weight analysis of ZnT${\;}_{3}^{-/-}$ and WT mice. Two-way ANOVA with LSD *post-hoc* test. **(C, D)** The blood glucose levels in male **(C)** and female **(D)** mice (ZnT${\;}_{3}^{-/-}$; and WT) after fasting; *n* = 6 for both ZnT${\;}_{3}^{-/-}$ and *n* = 5 for both WT groups. Two-way ANOVA with LSD *post-hoc* test. **(E, F)** mRNA expression levels of *Slc2a1* (GLUT1), *Slc2a3* (GLUT3), and *Slc2a4* (GLUT4) in cortex **(E)**; and *Slc2a1* (GLUT1), *Slc2a3* (GLUT3), and *Slc2a4* (GLUT4) and *Insr* (INSR) in hippocampus **(F)**. Expression levels are normalized to housekeeping gene *Gapdh*. * Denotes significant difference between groups indicated by the connector lines, *p* < 0.05, Mann-Whitney test. **(G, H)** Immunofluorescence staining of hippocampal sections of 9-months old mice. **(G)**: GLUT3-(red), DAPI-(blue) and GFAP (green). **(H)**: GLUT4-(green), DAPI-(blue) and NeuN (red). Scale bar, 50 μm; Zoom Scale bar, 5 μm. **(I, J)** Statistical analysis of images as those shown in **(G)** and **(H)**. * Denotes significant difference between groups indicated by the connector lines, *p* < 0.05, Mann-Whitney test.

To assess possible effect of ZnT_3_ deletion on metabolism, the weight of WT and ZnT_3_ knockout mice was monitored throughout first 9 months of age. There was a progressive increase in body weight among both male and female mice of both genotypes as they aged (Figure 5A). However, at 9 months of age, male ZnT${\;}_{3}^{-/-}$ mice exhibited slightly but significantly lower weight, compared to age-matched male WT controls (Figure 5A). The brain weight of ZnT${\;}_{3}^{-/-}$ mice (at 9 months) was found to be comparable to that of WT controls, suggesting that the absence of this gene does not significantly affect general brain development (Figure 5B). The blood glucose testing indicated that the blood glucose levels in ZnT${\;}_{3}^{-/-}$ mice of either gender (at 9 months of age) was comparable to these of their gender- and age-matched WT controls (Figures 5C, D). Taken together, even despite a small difference in bodyweight of aged male mice, the ZnT_3_ deletion does not seem to affect general metabolism and brain weight significantly.

Insulin resistance is a prevalent characteristic, observed in the AD brain. Zinc, an essential trace element, plays a significant role in insulin receptor-related functions (Li, 2013). Consequently, we explored potential correlation between the compromised behavioral outcomes and dendritic plasticity in the hippocampus of ZnT${\;}_{3}^{-/-}$ mice and the insulin signaling in the brain. First, we assessed the gene expression levels of glucose transporters, *Slc2a1* (GLUT1), *Slc2a3* (GLUT3), and *Slc2a4* (GLUT4), as well as the insulin receptor *Insr* (INSR) in the hippocampi and cortexes of 9-month-old mice using qPCR. Following the knockout of ZnT_3_, a significant decrease in the mRNA levels of *Slc2a3* was observed in the cortex. In hippocampus expression of *Slc2a3, Slc2a4*, and *Insr* was significantly decreased in 9-months-old ZnT${\;}_{3}^{-/-}$ mice (Figures 5E, F). In addition, we found that the genes encoding postsynaptic density protein 95, PSD95 (*Dlg4*), as well as AMP-activated protein kinase, AMPK (*Prkaa1*) and AKT (*Akt*) were also significantly reduced in the hippocampi of ZnT${\;}_{3}^{-/-}$ mice (Supplementary Figures 4A, B). For all the expression data, male and female mice were all used.

Immunofluorescence and western blot analysis was then used to asses potential changes in protein levels of GLUT3 and GLUT4. Immunofluorescence analysis revealed a significant decrease in the immunoreactivity of both GLUT3 and GLUT4 in CA1, CA3, and DG of the ZnT${\;}_{3}^{-/-}$ mice hippocampi, when compared to the control (WT) group (Figures 5G–J). GLUT4 immunoreactivity was mostly confined to neurons (identified by shape and NeuN expression; Figure 5H); GLUT3 was found both in neurons and in astrocytes (identified by GFAP; Figure 5G). Additionally, the fluorescence intensity of GLUT3 in the cortex of ZnT${\;}_{3}^{-/-}$ mice was also reduced, whereas the expression of GLUT4 did not change significantly (Supplementary Figures 4C–F). Western blot results indicated a decrease in the expression levels of GLUT3 in the neuronal “synaptosome” membrane fraction in the whole brain tissue of ZnT${\;}_{3}^{-/-}$ mice, with no significant change in the expression of GLUT4 (Supplementary Figures 5A–C).

**Figure 6:**
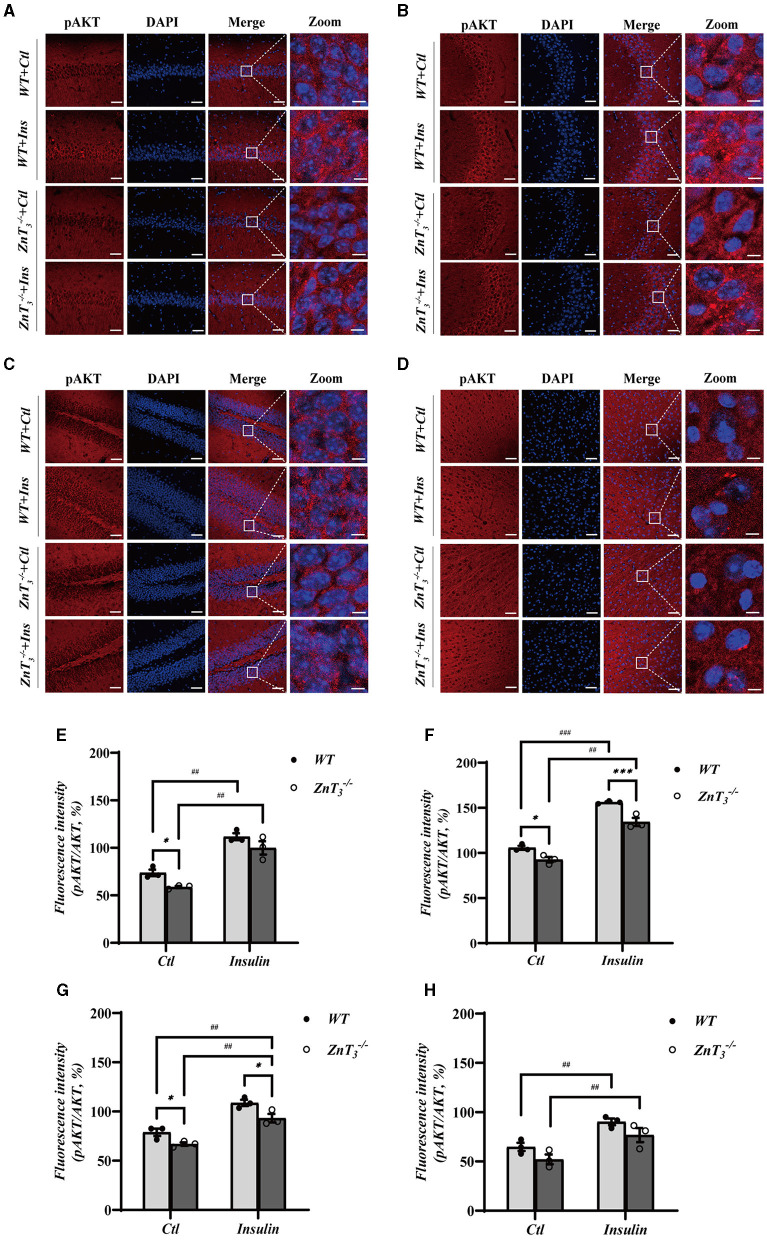
Deletion of ZnT_3_ impairs insulin-related signaling in hippocampus. **(A–D)** Immunofluorescence staining of hippocampal sections of 9-months old mice after intraperitoneal injection of insulin glargine (2 IU/kg i.p.; see Methods for detail). pAKT-(red), DAPI-(blue). **(A)**: CA1, **(B)**: CA3, **(C)**: DG, and **(D)**: cortex. Scale bar, 50 μm; Zoom Scale bar, 5 μm. **(E–H)** Statistical analysis of images as those shown in **(A–D)**. *, *** and ^##^, ^###^ Denote significant difference between groups indicated by the connector lines, *p* < 0.05, *p* < 0.01, or *p* < 0.001 (for single, double and triple symbols, respectively) two-way ANOVA with LSD *post-hoc* test.

To further investigate potential role of ZnT_3_ in the insulin signaling, we tested the effect of insulin administration on the downstream targets of insulin signaling, AKT and Glycogen synthase kinase 3β (GSK3β) expression in the hippocampus and cortex of WT and ZnT${\;}_{3}^{-/-}$ mice (9 months of age) using Western blot and immunofluorescence. There was no difference in the expression of GSK3β in the hippocampi of WT and ZnT${\;}_{3}^{-/-}$ mice and GSK3β levels were not affected by insulin (2 IU/kg insulin glargine; i.p. injection after 5 h fasting; analyzed 24 h after administration; Supplementary Figures 6A, B). Interestingly, while total levels of AKT immunoreactivity in hippocampi and cortexes was not different between the genotypes (Supplementary Figures 6C–J), the levels of pAKT was significantly reduced in the hippocampus (across all regions); there was no significant difference in cortex (Figure 6). Following insulin treatment, the levels of pAKT and pAKT/AKT ratio increased in hippocampus and cortex of animals of either genotype, however, even after insulin administration, ZnT${\;}_{3}^{-/-}$ mice had significantly lower pAKT levels in hippocampus, as compared to WT mice (Figure 6).

These data presented above demonstrate that ZnT_3_ deletion is associated with significant disruption in the insulin signaling pathway within the hippocampus and, to a lesser degree, in the cortex.

## Discussion

Zinc is abundantly present in the hippocampus and has been reported to play a significant role in the regulation of spatial learning and memory (Ni et al., 2009; Yang et al., 2013; Sopian et al., 2015). Within the hippocampus, two major zinc handling proteins were reported: the synaptic transporter, ZnT_3_ and zing binding “metallothionein,” MT_3_ (Prakash et al., 2015; Hojyo and Fukada, 2016; Kambe et al., 2021). ZnT_3_ plays a crucial role in the storage of zinc within synaptic vesicles of glutamatergic neurons, furthermore, the synaptic release of zinc was shown to produce an inhibitory influence on GABAergic synapses through feedforward inhibition, thereby facilitating long-term potentiation in amygdala (Kodirov et al., 2006).

Here we confirm that the deletion of ZnT_3_ results in a decrease in zinc levels in hippocampus and cortex. We show that this zinc deficiency is associated with spatial learning and memory dysfunctions, which are progressing with aging. Thus, new object recognition was significantly impaired in ZnT${\;}_{3}^{-/-}$ mice of either sex by the 9 months of age; likewise, aged animals performed worse in the Y-maze test. Additionally, 2-month-old mice showed impaired learning and memory in the MWM test regardless of gender and it is likely that this type of memory performance would further deteriorate with age (although MWM testing becomes problematic in older animals). These findings collectively suggest progressive deficit in spatial memory.

Spatial memory is a type of memory that stores and processes spatial information, it can be divided into spatial working memory and spatial reference memory (Sharma et al., 2010). Spatial working memory only lasts for a few seconds to a minute and can be tested in mice with such tests as novel object recognition and Y-maze (Mathiasen and DiCamillo, 2010; Kraeuter et al., 2019). Spatial reference memory can store a larger quantity of information and may have an indefinite duration and can be tested with MWM and T-maze tests (Dudchenko, 2004).

There is a body of evidence that young (3–10 weeks) ZnT${\;}_{3}^{-/-}$ mice have near-normal spatial learning and memory. Thus, Sindreu and colleagues (Sindreu et al., 2011) observed that male mice aged 3–4 weeks exhibited normal performance in the recognition of novel or relocated objects. Similarly, Cole et al. (2001) reported normal performance in a range of learning and memory tests, including MWM. These findings align well with the findings obtained in the present study from 1- and 2-month-old mice (male and female), showing normal performance in a range of spatial memory tests. Yet, previous investigations provided rather limited data on aged and female mice. Adlard et al. (2010) found a cognitive decline in 3-month-old ZnT${\;}_{3}^{-/-}$ mice (mixed-gender) using MWM testing and progressive decline in object location memory (OLM) at 3 and 6-months of age was also reported (also a mixed-gender study; Vogler et al., 2022). Here we monitored performance of male and female ZnT${\;}_{3}^{-/-}$ mice in NOR, NLR, MWM, Y-, and T-maze tests for up to 9 months of age. Our data suggest that while deficit in MWM performance is already apparent at 2 months (Figure 2), deficits in NOR and Y-maze performance are only beginning to manifest at a later stages of aging (i.e. at 9 months of age in the present study; Figure 1). The above deficits were equally prominent in male and female mice. It has to be noted that since MWM incorporates fear avoidance, it may assess spatial memory differently, as compared to various other tests that do not involve fear (Othman et al., 2022). This may explain the findings that deficits in MWM test performance were detected earlier than NOR and Y-maze test deficits. In sum, the behavioral tests performed here confirm near-normal spatial learning of young ZnT${\;}_{3}^{-/-}$ mice but reveal progressive deficits developing with aging.

We also found that ZnT_3_ deletion affected dendritic complexity and spine density in the hippocampus. After ZnT_3_ elimination, dendrites in hippocampal CA3 and DG regions become more complex, while dendritic spine density decreased. Apical dendrites and basal dendrites exhibit differences in characteristics such as electrical conduction and response to guiding molecules (Kaibara and Leung, 1993; Häusser et al., 2000; Arikkath, 2012). Here we show that ZnT_3_ knockout results in a decrease in dendritic spine density in both apical and basal dendrites.

Dendritic spines are small protrusions on the dendrites, and their highly diverse morphology is considered to be the basis of synaptic plasticity. Dendritic spines can be classified into four types based on their size and shape: mushroom, thin, stubby and branched (Peters and Kaiserman-Abramof, 1970). Large mushroom spines, may not have much capacity for further increase in synaptic strength, while new and thin spines which carry small or immature synapses, have higher potential for strengthening and are therefore considered to indicate a capacity for plasticity in the local circuit (Berry and Nedivi, 2017). Thus, large mushroom spines are referred to as memory spines, while thin spines as learning spines (Bourne and Harris, 2007). Stubby spines are mainly found on the dendritic shaft and rarely seen on the dendritic terminals; branched spines are not considered to play a role in synapse formation due to the lack of postsynaptic density (PSD) (Knott et al., 2006; Arellano et al., 2007), therefore these two types of spines are considered as immature (Berry and Nedivi, 2017).

There is limited data on the role of ZnT_3_ in the formation of various types of dendritic spines, however, there is a higher zinc content in the heads of the dendritic spines (Perrin et al., 2017). This is thought to be closely related to the formation of various types of dendritic spines, perhaps via zinc-dependent stabilization of tubulin (Craddock et al., 2012; Perrin et al., 2017). ZnT_3_ knockout was shown to affect hippocampal neuron plasticity in mice, and is also one of the important factors in dementia (McAllister and Dyck, 2017), which is consistent with findings showing the mice lacking ZnT_3_ exhibit reduced activation of key synaptic plasticity proteins (e.g., presynaptic MAPK signaling), leading to the synaptic impairment and memory deficits (Sindreu et al., 2011).

Importantly, insulin resistance in the brain also can damage hippocampal synaptic plasticity leading to abnormal synaptic function (Stranahan et al., 2008; Ansari et al., 2023). Morphological development of dendrites in hippocampal neurons is regulated by the brain-derived neurotrophic factor (BDNF) (Wang et al., 2015) and there are reports indicating that ZnT_3_ knockout mice show reduced BDNF expression (Vogler et al., 2022). However other studies reported that knocking out ZnT_3_ can lead to an increased levels of BDNF in the brain of younger mice, which was linked to increased dendritic length and more intricate connections between neurons (Helgager et al., 2014; Yoo et al., 2016; McAllister et al., 2020). Hence, the potential role of BDNF in ZnT_3_-dependent changes in dendritic complexity and spine density requires further investigation.

Recent research has increasingly associated family history of type 2 diabetes (T2DM) with AD (Thorpe et al., 2012; Futamura et al., 2015; Kawada, 2020). In addition, multiple studies have also found a close correlation between reduced brain glucose metabolism and the development of dementia (Kikuchi et al., 2011; Cukierman-Yaffe, 2014; Matthews et al., 2021). Moreover, deficiency in glucose metabolism can be considered as important early biomarkers of the AD; indeed this common characteristic of AD and type 1/2 diabetes was even recognized in the suggested term for AD as the “type 3 diabetes” (Ferreira S. T. et al., 2014; Michailidis et al., 2022).

GLUT3 is a glucose transporter protein largely responsible for neuronal glucose uptake. It is primarily distributed in neuronal processes, including dendrites, and expressed at high levels in presynaptic and postsynaptic nerve terminals (Koepsell, 2020). Additionally, a small amount of GLUT3 expression has been found in astrocytes (Simpson et al., 2008; Iwabuchi and Kawahara, 2011). Some studies suggest that peripheral insulin administration can induce synthesis of GLUT3 protein in the brain (Uehara et al., 1997). GLUT4 is expressed in multiple brain regions including the cerebral cortex, olfactory bulb, hypothalamus, and hippocampus, and mainly enriched in neurons, typically co-expressed with GLUT3 (Apelt et al., 1999; Choeiri et al., 2002; El Messari et al., 2002). In the absence of insulin, GLUT4 is primarily located within intracellular vesicles known as GLUT4 storage vesicles (GSVs) while insulin triggers its translocation to the plasma membrane (Du et al., 2017). It has been shown that GLUT4 plays an important role in the formation of synapses in the hippocampus (Ashrafi et al., 2017). Until now, the interaction between zinc and GLUT3/4 remains unknown.

Here we show that the transcript and protein levels of *Slc2a3* (GLUT3) and *Slc2a4* (GLUT4) in the CA1, CA3, and DG regions of the hippocampus were significantly reduced in ZnT${\;}_{3}^{-/-}$ mice when compared to the WT controls; the protein levels of GLUT3 in the whole-brain “synaptosome” membranes of knockout mice also decreased; in addition, insulin receptor gene transcript level was also significantly reduced in hippocampus (Figure 5, Supplementary Figures 4, 5). It is tempting to speculate that the absence of ZnT_3_ and decrease in synaptic zinc levels affects the expression and plasma membrane levels of GLUT3 and GLUT4 in the brain. In this way zinc can, in some way, mimic the function of insulin, as was already suggested (Wu et al., 2016). Indeed, zinc was shown to activate GLUT4 translocation into the plasma membrane and its phosphorylation by GSK3β, thereby increasing the glucose transport, and enhancing the cellular glucose uptake capacity (Vardatsikos et al., 2013; Wu et al., 2016; McNay and Pearson-Leary, 2020).

AKT is a major mediator of cellular effects of insulin (Lawlor and Alessi, 2001). Importantly, zinc was shown to trigger AKT phosphorylation in 3T3-L1 rat adipocytes, affecting the phosphorylation of GSK3β and promoting cellular glucose uptake through the translocation of GLUT4 from the cytosol to the plasma membrane (Basuki et al., 2007; Nakayama et al., 2008). Dietary zinc supplementation was also shown to enhance the phosphorylation of GSK3β (Baltaci et al., 2022). Here we reveal that in the hippocampi of ZnT${\;}_{3}^{-/-}$ mice, the total levels of AKT and GSK3β remains unchanged, but the pAKT levels decrease significantly (compared to WT animals). Following insulin treatment, the level of pAKT and pAKT/AKT ratio in the hippocampus and cortex increased in both WT and ZnT${\;}_{3}^{-/-}$ animals, although in the latter, the levels of pAKT and pAKT/AKT ratio were still lower than in the WT mice (Figure 6, Supplementary Figure 6). Phosphorylation of AKT affects the expression and membrane translocation of GLUT4 (Dugani and Klip, 2005; Khan and Kamal, 2019). Therefore, weaker effect of ZnT_3_ knockout on the pAKT levels in the cortex (as compared to hippocampus) may be the reason for the lack of reduction in GLUT4 expression in the cortexes of ZnT${\;}_{3}^{-/-}$ mice.

**Figure 7:**
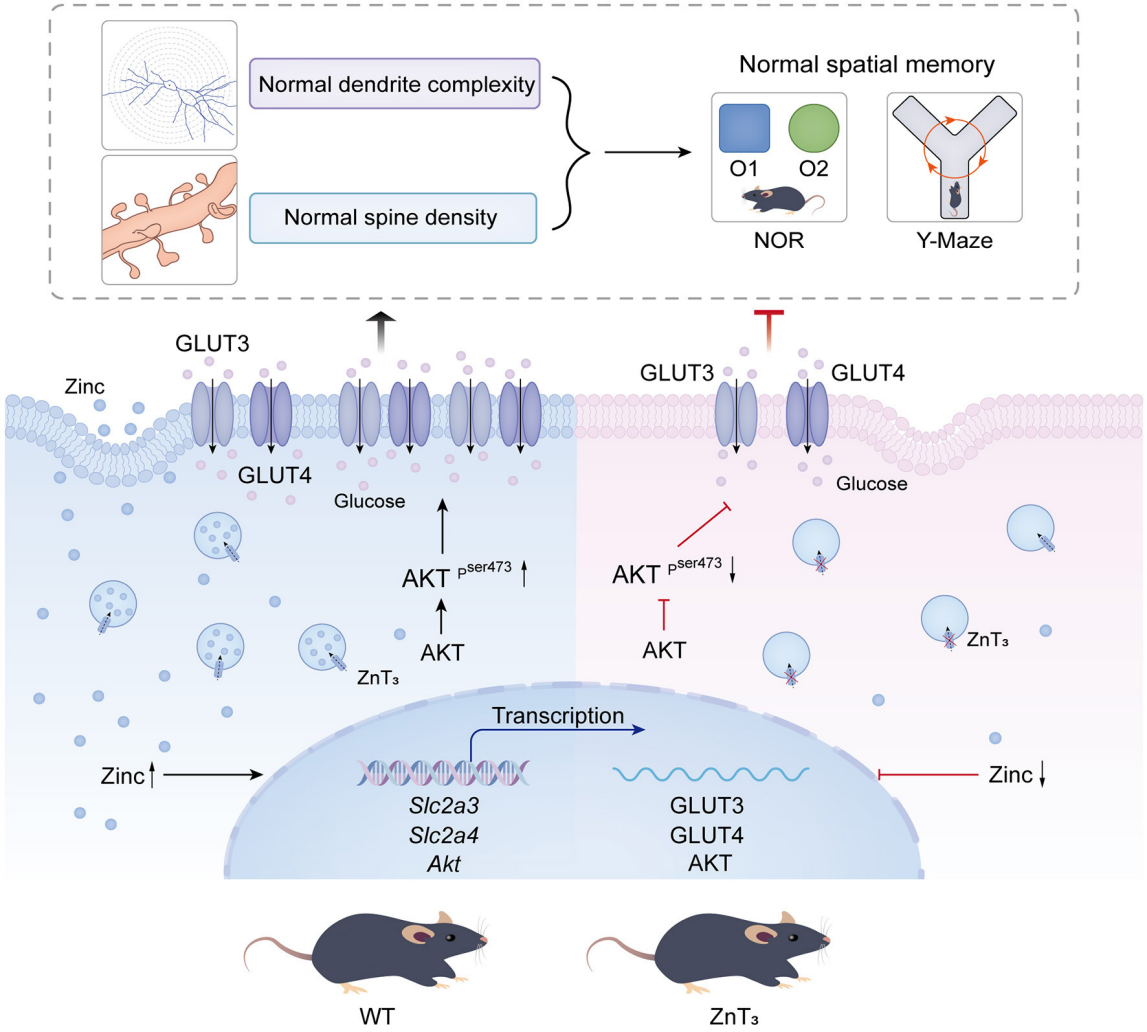
Summary of the observed and proposed effects of ZnT_3_ deficiency in the brain.

In sum, we hypothesize that multiple deficiencies in neuronal glucose handling machinery in hippocampi of ZnT${\;}_{3}^{-/-}$ mice may hinder the dendritic spine formation and underline cognitive deficits observed. Thus, our results suggest that ZnT_3_ knockout can cause series of pathological changes in glucometabolism, reminiscent of these of AD. These ideas are schematically depicted in Figure 7.

## Conclusions

The findings of this study demonstrate that the genetic deletion of ZnT_3_ and a reduction in synaptic zinc concentration within the hippocampus are associated with impaired spatial memory, which is exascerbated with age. Furthermore, a decline in the density of mature dendritic spines, particularly in hippocampus, was observed. Additionally, the examination revealed an augmentation in dendritic complexity within the hippocampal CA3 and DG regions and a significant increase in the overall length of dendrites in these neurons. Such increase in dendritic length and complexity could be a compensatory mechanism to offset the loss of spines. Finally, a decrease in molecules and pathways linked to insulin signaling was observed in both the hippocampal region and the cortex, which correlated with the reduction in dendritic spine density in hippocampal neurons and cognitive deficites.

## Data availability statement

The original contributions presented in the study are included in the article/Supplementary material, further inquiries can be directed to the corresponding authors.

## Ethics statement

The animal study was approved by the Animal Care and Ethical Committee of Hebei Medical University (approval number: IACUC-Hebmu-2020007). The study was conducted in accordance with the local legislation and institutional requirements.

## Author contributions

RZ: Investigation, Methodology, Project administration, Writing—original draft, Writing—review & editing. XZ: Investigation, Methodology, Writing—review & editing. XD: Investigation, Methodology, Writing—review & editing. GL: Investigation, Methodology, Writing—review & editing. JZ: Investigation, Methodology, Writing—review & editing. YG: Investigation, Methodology, Writing—review & editing. ZZ: Methodology, Writing—review & editing, Investigation. YM: Methodology, Writing—review & editing, Investigation. HG: Conceptualization, Funding acquisition, Methodology, Project administration, Supervision, Writing—original draft, Writing—review & editing. NG: Conceptualization, Funding acquisition, Methodology, Supervision, Visualization, Writing—original draft, Writing—review & editing.
