# Supplementary material for: Genetic deletion of zinc transporter ZnT3 induces progressive cognitive deficits in mice by impairing dendritic spine plasticity and glucose metabolism
Source: Front Mol Neurosci. 2024 May 14;17:1375925. doi: 10.3389/fnmol.2024.1375925 (PMC11130425; doi:10.3389/fnmol.2024.1375925)
Supplement: Supplementary file 1 [file Data_Sheet_1.docx]

**Genetic deletion of zinc transporter ZnT_3_ induces progressive cognitive deficits in mice by impairing dendritic spine plasticity and glucose metabolism**

**Supplementary data**

**Supplementary Table 1. Primer sets used for PCR reactions.**

| Gene | Primer sequence | Function |
| --- | --- | --- |
| *Slc30a3* Forward | 5′-ACCACCTTCCTCTTCTCTATCT-3′ | Target gene |
| *Slc30a3* Reverse | 5′-TCCCTTACAGGCTCAAATTCC-3′ | Target gene |
| *Slc2a1* Forward | 5′-ATGGATCCCAGCAGCAAGAAG-3’ | Target gene |
| *Slc2a1* Reverse | 5′-AGAGACCAAAGCGTGGTGAG-3’ | Target gene |
| *Slc2a3* Forward | 5′-GGATCCCTTGTCCTTCTGCTT-3’ | Target gene |
| *Slc2a3* Reverse | 5′-ACCAGTTCCCAATGCACACA-3’ | Target gene |
| *Slc2a4* Forward | 5′-CGGCTCTGACGATGGGGAA-3’ | Target gene |
| *Slc2a4* Reverse | 5′-TTGTGGGATGGAATCCGGTC-3’ | Target gene |
| *Insr* Forward | 5′-CTGAAGGAGCTGGGTCTTTAC-3′ | Target gene |
| *Insr* Reverse | 5′-GCCAGGTAGCAGAGTTCATTAT-3′ | Target gene |
| *Dlg4* Forward | 5′-ACGAGAGTGGTCAAGGTTAAAG-3′ | Target gene |
| *Dlg4* Reverse | 5′-GATGATGATGGGACGAGCATAG-3′ | Target gene |
| *Prkaa1* protein kinase Forward | 5′-GTGAAGATCGGCCACTACATCC-3’ | Target gene |
| *Prkaa1* protein kinase Reverse | 5′-GGCTTTCCTTTTCGTCCAACC-3’ | Target gene |
| *Akt* Forward | 5′-TCACGTGAGCCCTTCTCCTA-3’ | Target gene |
| *Akt* Reverse | 5′-CTCCCACCCACTAACAAGGC-3’ | Target gene |
| *Gapdh* Forward | 5′-GGGCCAAAAGGGTCATCATC-3′ | Reference gene |
| *Gapdh* Reverse | 5′-AACCTGGTCCTCAGTGTAGC-3′ | Reference gene |

**Supplementary Figure 1. Confirmation of ZnT_3_^-/-^ knockout and hippocampal zinc level reduction.**

(A) Diagram of ZnT_3_ knockout strategy (based on Cole et al. 1999). No, *NotⅠ*; Nh, *NheⅠ*; *nlac Z*, reporter gene; *neo^r^*, neomycin resistance gene.

(B) Example of PCR genotyping of DNA extracted from the brain of WT, ZnT_3_^+/-^ and ZnT_3_^-/-^ mice.

(C) The mRNA expression of *Slc30a3* (ZnT_3_) in the hippocampus of WT and ZnT_3_^-/-^ mice. n=3. Here and everywhere mean data are presented as mean ± S.E.M. ^*^ Denotes significant difference between groups indicated by the connector lines, p<0.05, Mann-Whitney test.

(D) Immunofluorescence staining of hippocampal sections from the WT and ZnT_3_^-/-^ mice. ZnT_3_-(red), DAPI-(blue) Hippocampus: scale bar, 200 μm; CA3 and DG: Scale bar, 50 μm.

(E) Immunofluorescence staining of cortex sections from the WT and ZnT_3_^-/-^ mice. ZnT_3_-(red), DAPI-(blue) Cortex: scale bar, 50 μm; Zoom: Scale bar, 20 μm.

(F) TSQ staining of Zn^2+^ in hippocampus sections from the WT and ZnT_3_^-/-^ mice. Scale bar, 200 μm

(G) Timm staining of the hippocampus sections from the WT and ZnT_3_^-/-^ mice. Hippocampus: scale bar, 500 μm; CA1-DG: scale bar, 200 μm.

**Supplementary Figure 2. ZnT_3_ knockout does not affect the performance in the T-maze assay.**

(A) Schematic diagram of T-maze.

(B)-(C) Analysis of the T-maze experiments: learning phase (B), reverse learning phase (C). WT and ZnT_3_^-/-^ groups, n=4. Kruskal-Wallis ANOVA.

**Supplementary Figure 3. ZnT_3_ deletion is associated with reduced basal and apical CA1 spine density in the hippocampus.**

(A) Diagram showing locations of basal and apical segments of a CA1 neuron taken for spine density analysis. Scale bar, 50 μm.

(B) Example Golgi staining images of dendritic spines of basal and apical segments in CA1 regions of hippocampi of 1-month-old, 2-months-old and 9-months-old mice. Scale bar, 5 μm.

(C) Statistical analysis of dendritic spine density from images as these shown in B in CA1 region of hippocampus. ^*^, ^**^, ^***^, ^****^ and ^#^, ^##^, ^###^, ^####^ Denote significant difference between groups is indicated by the connector lines, p<0.05, p<0.01, p<0.001 or p<0.0001, two-way ANOVA with LSD post-hoc test.

(D)-(G) Statistical analysis for Golgi staining images of different types of spines from basal and apical segments of CA1 neurons. Mushroom (D), stubby (E), thin (F) and branched (G) spines were from two- and nine-month-old mice (male and female) were analyzed. ^*^, ^**^, ^***^, ^****^ Denote significant difference between groups indicated by the connector lines, p<0.05, p<0.01, p<0.001 or p<0.0001, two-way ANOVA with LSD post-hoc test.

**Supplementary Figure 4. Impact of ZnT_3_ deletion on the expression of genes related to synapse formation and metabolism.**

(A)-(B) mRNA levels of *Dlg4* (PSD95), *Prkaa1* protein kinase (AMPK) and *Akt* (AKT) in cortex (A) and hippocampus (B). Expression levels are normalized to housekeeping gene *Gapdh*. ^*^ Denotes significant difference between groups indicated by the connector lines, p<0.05, Mann-Whitney test

(C)-(D) Immunofluorescence staining of cortical sections of 9-month-old WT and ZnT_3_^-/-^ mice. (C): GLUT3-(red), DAPI-(blue) and GFAP (green). (D): GLUT4-(green), DAPI-(blue) and NeuN (red). Scale bar, 50 μm; Zoom Scale bar, 5 μm.

(E)-(F) Statistical analysis of images as those shown in (C) and (D). ^*^ Denotes significant difference between groups indicated by the connector lines, p<0.05, Mann-Whitney test.

**Supplementary Figure 5. Impact of ZnT_3_ deletion on the expression of GLUT3 in synaptosome membrane fraction of the whole brain.**

(A) Example western blot detection of GLUT3, GLUT4 and GAPDH in the whole brain synaptosomes of 9-months-old mice.

(B)-(C) Statistical analysis of images as those shown in (A). GLUT3 (B) and GLUT4 (C). ^*^, ^**^, ^***^, ^****^ Denote significant difference between groups indicated by the connector lines, p<0.05, p<0.01, p<0.001 or p<0.0001, Independent-Samples T test.

**Supplementary Figure 6. Deletion of ZnT_3_ affects insulin signalling.**

(A) Example western blot detection of GSK3β blots in hippocampus of 9-months-old mice after intraperitoneal injection of insulin (see Methods for detail).

(B) Statistical analysis of images as those shown in (A).

(C)-(F) Immunofluorescence staining of hippocampal sections of 9-months old mice after intraperitoneal injection of insulin. AKT-(red), DAPI-(blue). (C): CA1, (D): CA3, (E): DG and (F): cortex. Scale bar, 50 μm; Zoom Scale bar, 5 μm.

(G)-(J) Statistical analysis of images as those shown in (C)-(F). Two-way ANOVA with LSD post-hoc test.
