# Supplementary figures and images for: Genetic deletion of zinc transporter ZnT3 induces progressive cognitive deficits in mice by impairing dendritic spine plasticity and glucose metabolism
Source: Front Mol Neurosci. 2024 May 14;17:1375925. doi: 10.3389/fnmol.2024.1375925 (PMC11130425; doi:10.3389/fnmol.2024.1375925)

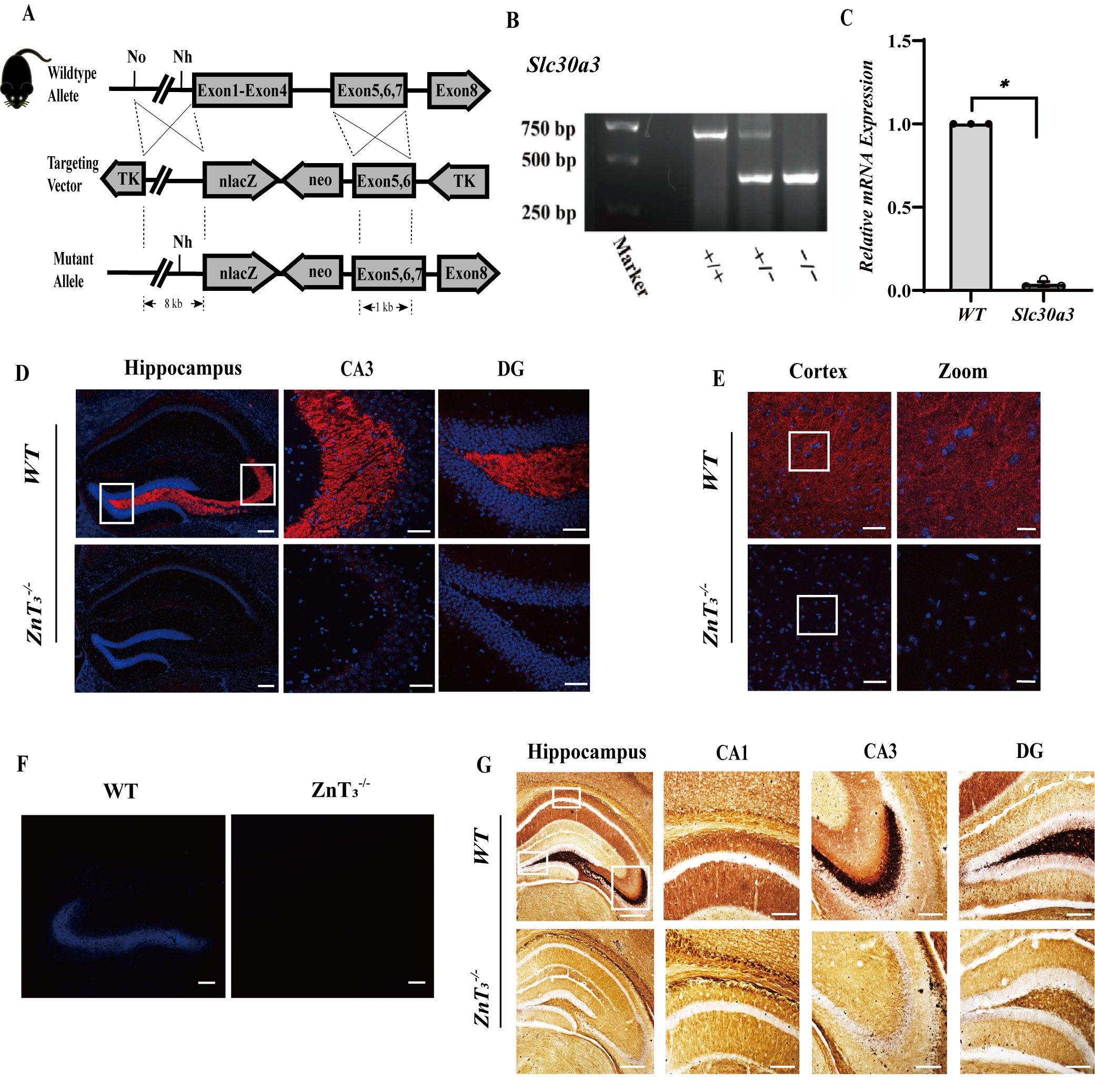

Supplement: Supplementary file 2 [file Image_1.jpeg]

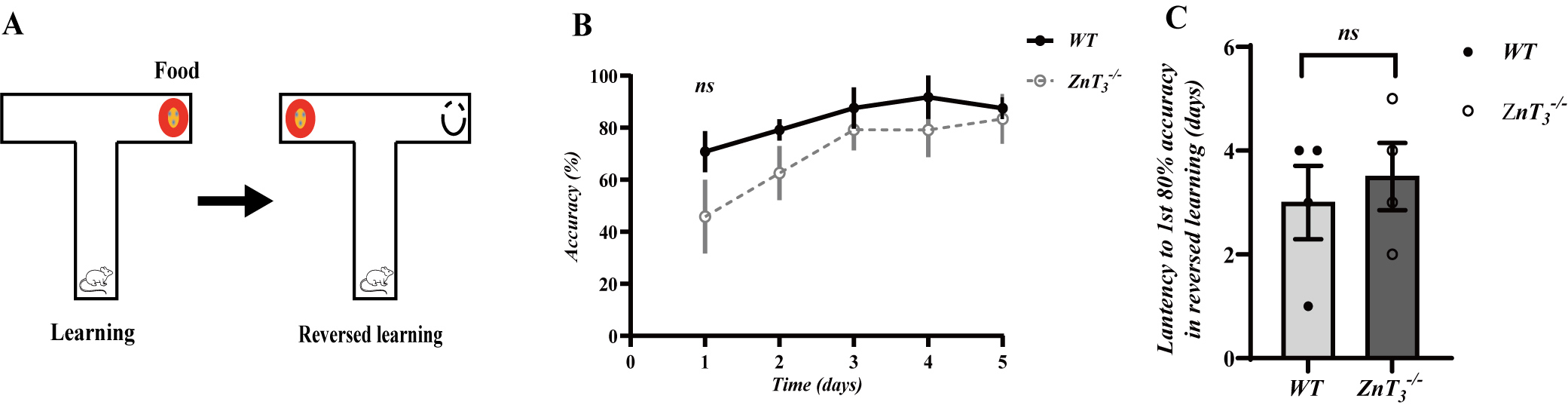

Supplement: Supplementary file 3 [file Image_2.jpeg]

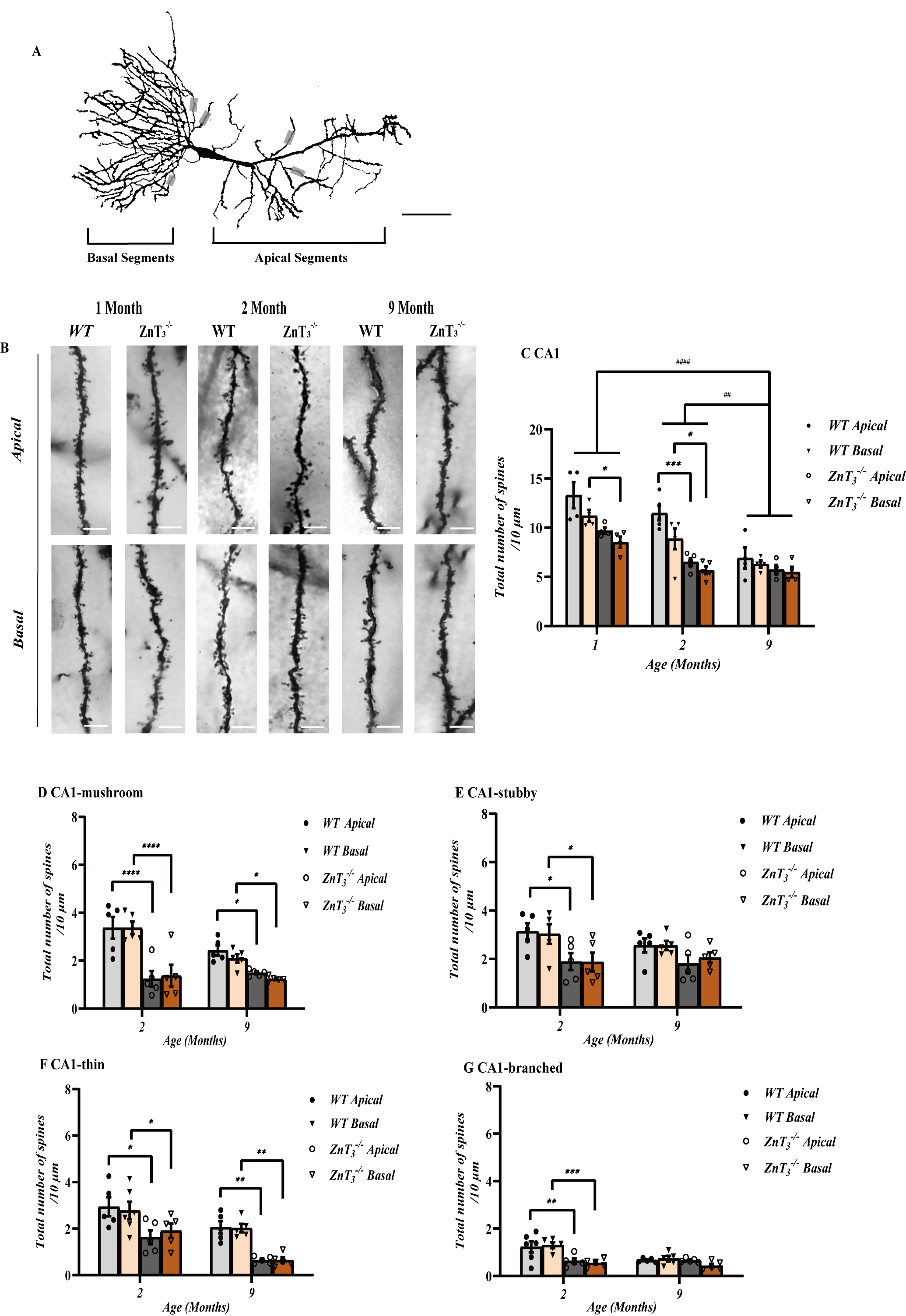

Supplement: Supplementary file 4 [file Image_3.jpeg]

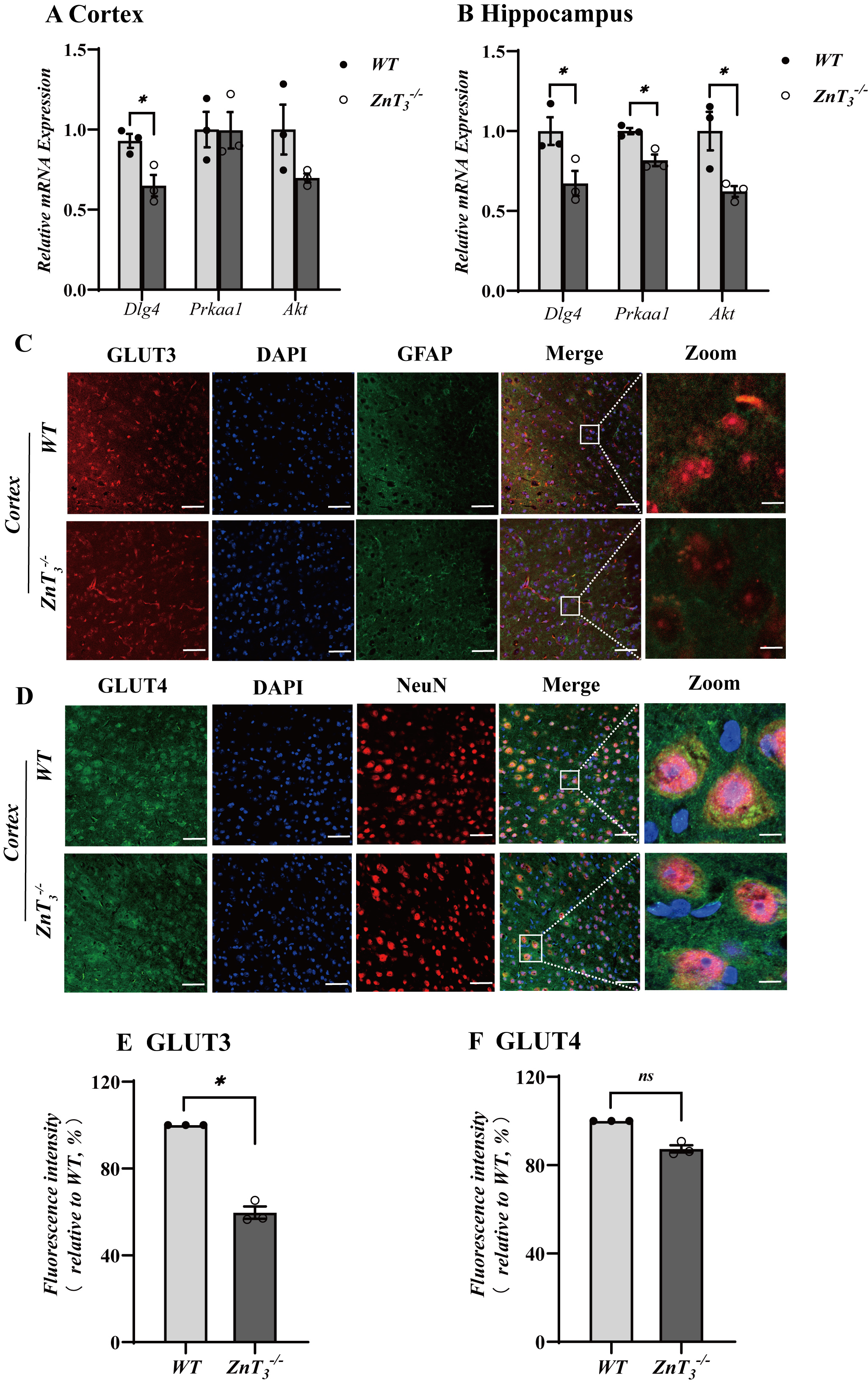

Supplement: Supplementary file 5 [file Image_4.jpeg]

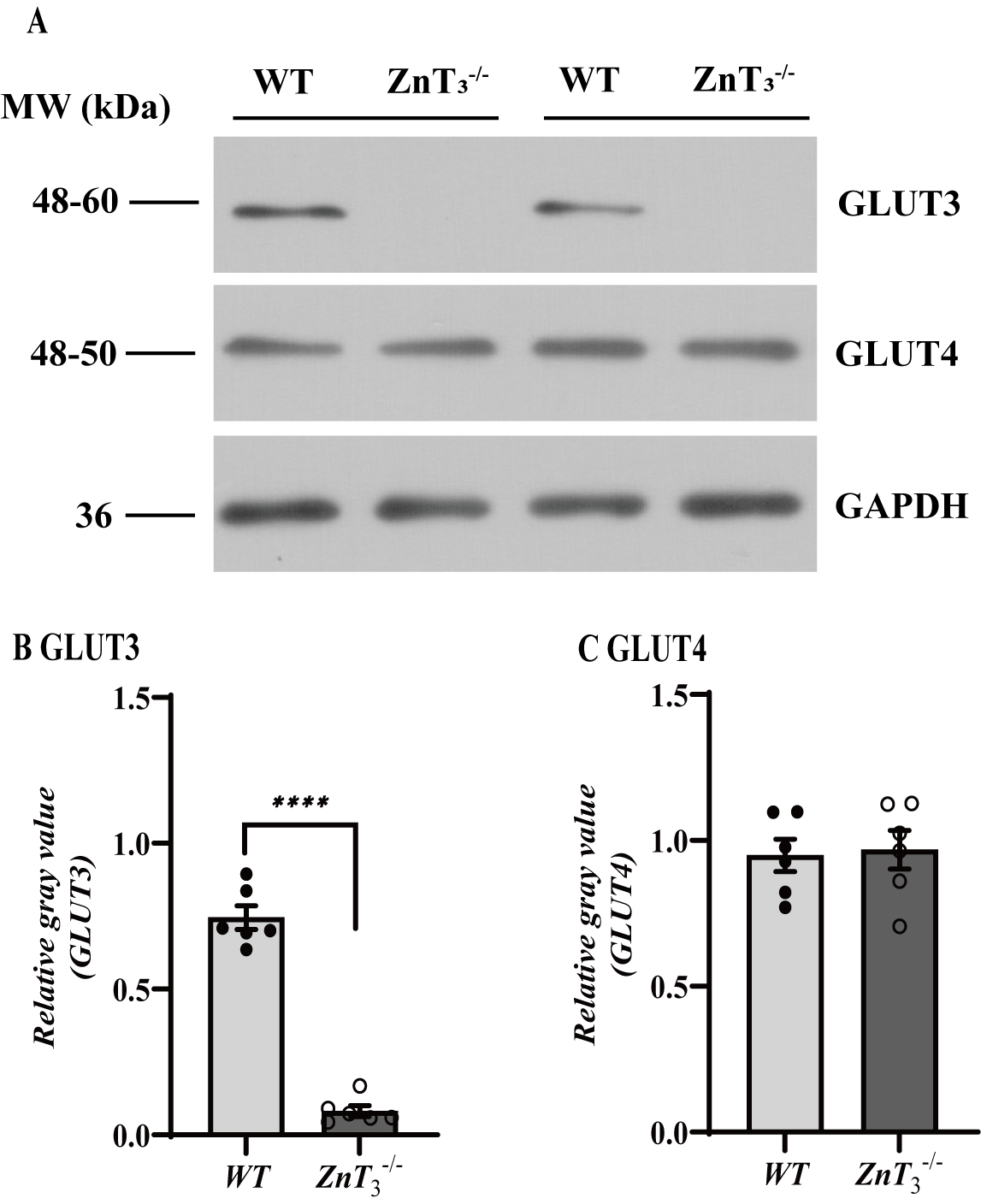

Supplement: Supplementary file 6 [file Image_5.jpeg]

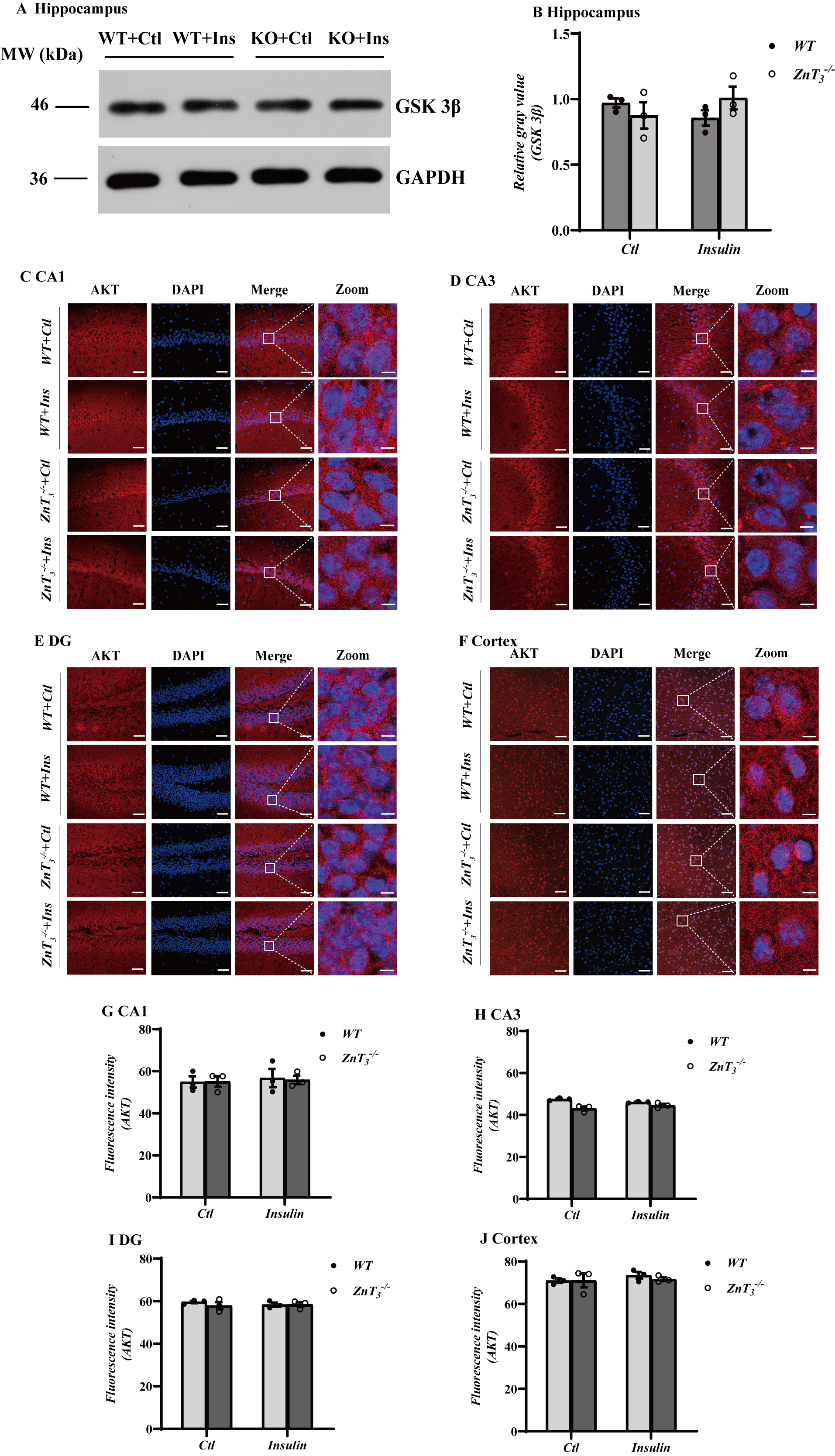

Supplement: Supplementary file 7 [file Image_6.jpeg]
